# Trapped in Place? Ethnic and Educational Heterogeneity in Residential Mobility and Integration of Young Adults in Brussels

**DOI:** 10.1007/s10680-023-09690-3

**Published:** 2024-01-25

**Authors:** Lena Imeraj, Sylvie Gadeyne

**Affiliations:** 1 Brussels Centre for Urban Studies (BCUS), Cosmopolis Centre for Urban Research, Interface Demography, Vrije Universiteit Brussel, Brussels, Belgium; 2 Interface Demography, Vrije Universiteit Brussel, Brussels, Belgium

**Keywords:** Residential mobility, Education, Ethnic background, Intergenerational, Segregation, Brussels

## Abstract

Spatial assimilation theory asserts that immigrants’ socioeconomic progress leads to residential adaptation and integration. This association has proven robust in USA and European urban areas through much of the twentieth century, but drastic change of ethnic and class compositions yet persistent (neighbourhood) inequality in the urban landscape urge us to reconsider the dynamic interaction between stability and change. In this study, we investigate to what extent education shapes residential mobility differently for young adults with varying ethnic and social origins. Focussing on Brussels, we use multinomial logistic regressions on linked longitudinal population-based censuses from 1991 and 2001 and register data for the period 2001–2006. Analyses show that dispersal away from poor inner-city neighbourhoods appears least likely for the offspring of poor low-educated non-Western households, regardless of their own educational attainment. While our approach roughly confirms traditional arguments of socio-spatial integration, it also reveals how educational success generates opportunities to escape poor neighbourhoods for some but not for others. With this, it points at the subtle ways in which factors and mechanisms in traditional spatial assimilation theory affect residential behaviour of young adults over their life course, at the intersection of specific locales, ethnic groups, social classes and generations.

**Supplementary Information:**

The online version contains supplementary material available at 10.1007/s10680-023-09690-3.

## Introduction

Several decades of migration to Europe have led to rapidly changing urban populations and increasingly complex urban geographies (Arbaci, 2019; Skifter Andersen, 2019). Today, many of the receiving societies struggle with demographic change, ethnic diversification and residential segregation of migrant populations (Piekut et al., 2019; Smith, 2019). Despite trends of de-segregation, reflected in decreasing native-dominated neighbourhoods and increasing prevalence of multi-ethnic neighbourhoods (Benassi et al., 2023; Catney et al., 2021, 2023), many highly segregated spaces persist (Andersson et al., 2018; Imeraj et al., 2018a), spurring lively public and policy debates on (im)migrant integration and segregation. Empirical work anchored in traditional theories of immigrant spatial assimilation, ethnic enclave/conflict and place stratification has documented levels and patterns of ethnic residential segregation as well as the determinants and processes that underlie and (detrimental) consequences that evolve from these spatial configurations (Boterman et al., 2021; Galster & Sharkey, 2017; Kaupinnen & van Ham, 2019). More recently, this body of research has shifted its focus from conventional explanations like socioeconomic disparities, individual preferences, and discrimination to delve into the intricate interplay of these factors within the multistep residential sorting process. Various new frameworks to study segregation have been put forward, in Europe, the USA and beyond, ranging from the *social structural sorting perspective* and *cycle of segregation* by Krysan and Crowder (2017), *perpetuation theory* by Goldsmith (2016), *spatial opportunity structures* by Galster and Sharkey (2017), and a *vicious circle of segregation* (Tammaru et al., 2021), a *domains* (van Ham & Tammaru, 2016) or *multiple-context* approach (Park & Kwan, 2017). These new frameworks all have in common the explicit focus on intersecting temporal and contextual dimensions, moving beyond the mere focus on static and single-scale measurements of segregation and underscoring the intricate determinants and selection mechanisms in different life domains and over generations, crucial in understanding why levels and patterns of segregation have been slow to change. The persistence of residential segregation of the rich and the poor in European and American cities (Haandrikman et al., 2023; Quillian & Lagrange, 2016; Tammaru et al., 2016), the overlap of socioeconomic and ethnic concentration patterns (Costa & de Valk, 2018a; Harris et al., 2017), and the similarity of socioeconomic and ethnic contexts of individuals and their parents (Gustafsson et al., 2017; Pais, 2017; Sharkey, 2008, 2013), clearly illustrate how processes and outcomes of socioeconomic and ethnic sorting intersect over (biographic and processual) time, having important repercussions for the future of social and ethnic stratification of European society and beyond.

Previous studies show that ethnic minorities tend to settle or be trapped in certain neighbourhoods and housing market segments, by choice or by lack of choice, whereas native-born majority households refrain from settling in or escape from these neighbourhoods (Andersson, 2013; Bolt et al., 2008; Bråmå, 2006; Clark & Coulter, 2015). While residential sorting processes can be related to the persistent ethnic gap in educational attainment, and the differential residential opportunities and outcomes associated herewith (Bailey, 2012; Heath & Brinbaum, 2007), this association does not explain why ethnic minorities, even after attaining higher education, still exhibit a greater likelihood of residing in deprived neighbourhoods (Bolt & van Kempen, 2010; Boschman et al., 2017; de Vuijst et al., 2017; Kalm et al., 2023). This suggests education to have a different impact on residential behaviour, being moderated and collectively shaped by various individual characteristics, household factors and the broader socioeconomic context in which individual lives are embedded (Bernard & Vidal, 2019). Accordingly, this paper argues that instead of merely examining the influence of educational attainment—as one dimension of socioeconomic status—on residential mobility and attempting to separate its effects from non-racial/ethnic factors and intergenerational transmission mechanisms, it is crucial to dedicate more effort to understanding the complex interplay of various factors at the individual, household, and neighbourhood levels. By adopting a comprehensive approach that considers life course, linked lives, community, and context, we aim to emphasise ways in which the *triple inequality*, i.e. the close relationship between spatial, socioeconomic/educational, and ethnic inequality (Andersson & Kährik, 2016), unfolds across generations to perpetuate urban inequalities.

The current paper uses nationwide longitudinally linked 1991 and 2001 Census data and Register data on internal migration between 2001 and 2006 to examine selective residential mobility. The empirical analysis reported here focuses on individuals living in deprived and ethnic dense inner-city areas in the Brussels-Capital Region (BCR) in the period 1991–2006. By questioning how education, both self-attained and inherited, influences distinct residential patterns in different ethnic communities in BCR, our contribution to contemporary understandings of socio-spatial inequality is threefold. First, our study considers *linked lives* by adopting an intergenerational perspective that explores whether the role of educational attainment in moving is mitigated by parental education. Second, our study ruminates *intersectionality* by delving into ethnic variability in socio-spatial trajectories. Third, our data are *individual* and *longitudinal*, allowing for a dynamic and mobility-based exploration of segregation. These insights provide a deeper understanding of the heterogeneity in educational returns to residential moving behaviour in the Brussels context, and hence residential sorting mechanisms underpinning urban change.

## Residential Sorting and Educational Attainment: Theorising Ethnic and Intergenerational Heterogeneity

Our study adopts a life course perspective, with a particular focus on transitions into adulthood, defined as critical shifts in social roles or status—between the early twenties and thirties—, such as leaving home, completing education, entering full-time employment, building a family or becoming a parent, all of which tend to be associated with upward or downward moves in the neighbourhood poverty distribution (Bernard et al., 2014; Brazil & Clark, 2018). As young adults’ residential moves have the potential to perpetuate residential segregation (Britton & Goldsmith, 2013), our study specifically focuses on selective residential changes and neighbourhood attainment as part of life course transitions (Coulter et al., 2016). A key inquiry regarding neighbourhood attainment revolves around the concept of ‘locational returns’ (Logan & Alba, 1993), which questions whether the acquisition of human capital and socioeconomic advancement affords second-generation immigrants the opportunity to access better neighbourhood environments comparable to those of native-born individuals. Relying on the notion of a *vicious circle of segregation* (Tammaru et al., 2021), we explore various conceptual and theoretical frameworks as well as empirical evidence on the potential (causal) pathways through which unequal spatial patterns arise, giving particular attention intergenerational and ethnic variance in educational selection and sorting of young adult mobilities.

Founded in the Chicago School's ecological model of urban immigrant incorporation and bringing together residential segregation and mobility, *spatial assimilation theory* contends that socioeconomic progress of migrants (offspring) steadily results in a dispersal away from densely populated (co-)ethnic concentrations (Massey, 1985). The notion of associated socio-spatial modification encircles two residential mobility paths, that is, moves from poor ethnic dense inner-city locations to urban areas with more well-off natives and moves from inner-city concentrations towards suburban destinations (Massey, 1985). Extensive investigation into both pathways has revealed that a good number of social climbers depart from poor native-scarce urban areas to wealthier native-dense areas, urban as well as suburban (Alba & Nee, 1997; Bolt & van Kempen, 2010; Musterd et al., 2016; Simpson & Finney, 2009). While such spatial adaptation is assumed to be a response to reduce social distance (Musterd et al., 2016; van Gent et al., 2019), residential change appears less likely among non-Western young adults than among native and Western counterparts (Bolt & van Kempen, 2010; Boschman & van Ham, 2015) and consolidates the residential attainment gap between highly educated people of Western- and non-Western origin (de Vuijst et al., 2017). Ethnic minorities’ restricted spatial integration in part is involuntary because they are excluded from residing in ‘desirable’ areas as they face more external constraints in their work and housing trajectories, potentially disposing of fewer financial and non-financial resources or being confronted with structural constraints or discrimination in the housing market, as argued in the *place stratification perspective* (Alba & Logan, 1993; Bolt & van Kempen, 2003; Boschman et al., 2017). In anticipation of possible exclusion, discriminated or disadvantaged mobile residents are shown to stay within a limited radius of their origin neighbourhoods as they prefer to live with co-ethnics to benefit from the closeness of kinship, social ties, community-based resources and housing opportunities (Musterd et al., 2016; Spring et al., 2017; Van der Laan Bouma-Doff, 2007). This *self-segregation* emphasises voluntary factors and preferences aimed at social or ethnic homogeneity in shaping unequal residential attainments/geographies. The most speaking example of residential socio-ethnic homophily is observed in the avoidance of and to a lesser extent flight from poor migrant areas by natives (Skifter Andersen, 2017). Evidently, the spatial avoidance or neighbourhood attachment of a particular population group, minority or majority, is driven by heterogeneous preferences, going from fear of crime or religious fundamentalism to a desire to preserve the own-group, escape from ethnic prejudice and search for belonging and trust (Finney & Simpson, 2009; Voas & Fleischmann, 2012). Based on the place stratification and self-segregation perspectives, minority residents may thus face a double disadvantage, namely in terms of their ethnic status and resource status.

The accumulation of neighbourhood deprivation across generations, particularly for those from non-Western origin (de Vuijst et al., 2017; Hedman et al., 2013; van Ham et al., 2014) thus strongly suggests that ending up in the most advantageous social and residential status remains in favour of those with the most privileged and native background. Relevant in this respect is the *vicious* interaction between individual and place over time. On the one hand, neighbourhoods with poor resources are assumed to give rise to fewer opportunities for learning and to lower education, hence fewer (future) opportunities for out-migration (Galster, 2012; Galster & Sharkey, 2017). On the other hand, processes of population sorting that define the social and ethnic neighbourhood make-up—and thus available resources—are determined by intergenerational household (parental) resources and ethnic background (Bailey et al., 2017; Gustafsson et al., 2017; Hermansen et al., 2022; Hostenbach, 2018; Pais, 2017, 2021). Given the differential distribution of parental education across and within ethnic groups and the differential sorting mechanisms generated hereby, this study hypothesises that the role of individual educational attainment in residential behaviour is mitigated by parental and neighbourhood contexts differently in ethnic group populations.

To date, we do not dispose of sound knowledge regarding the joint impact of achieved and inherited human capital forms on young adults’ residential behaviour, both migrant- and native-origin, to whom secure housing arrangements have become increasingly unattainable (Hostenbach, 2018; Van Criekingen, 2009). This study investigates micro-level associations between education and residential mobility outcomes, accounting for social and ethnic origins and resources. Hence, it shifts from a general cross-sectional framework of socio-spatial integration towards a longitudinal, group-specific and intergenerational perspective that emphasises the role of social and ethnic background in creating uneven spatial outcomes in urban populations.

## Brussels Context

**Fig. 1:**
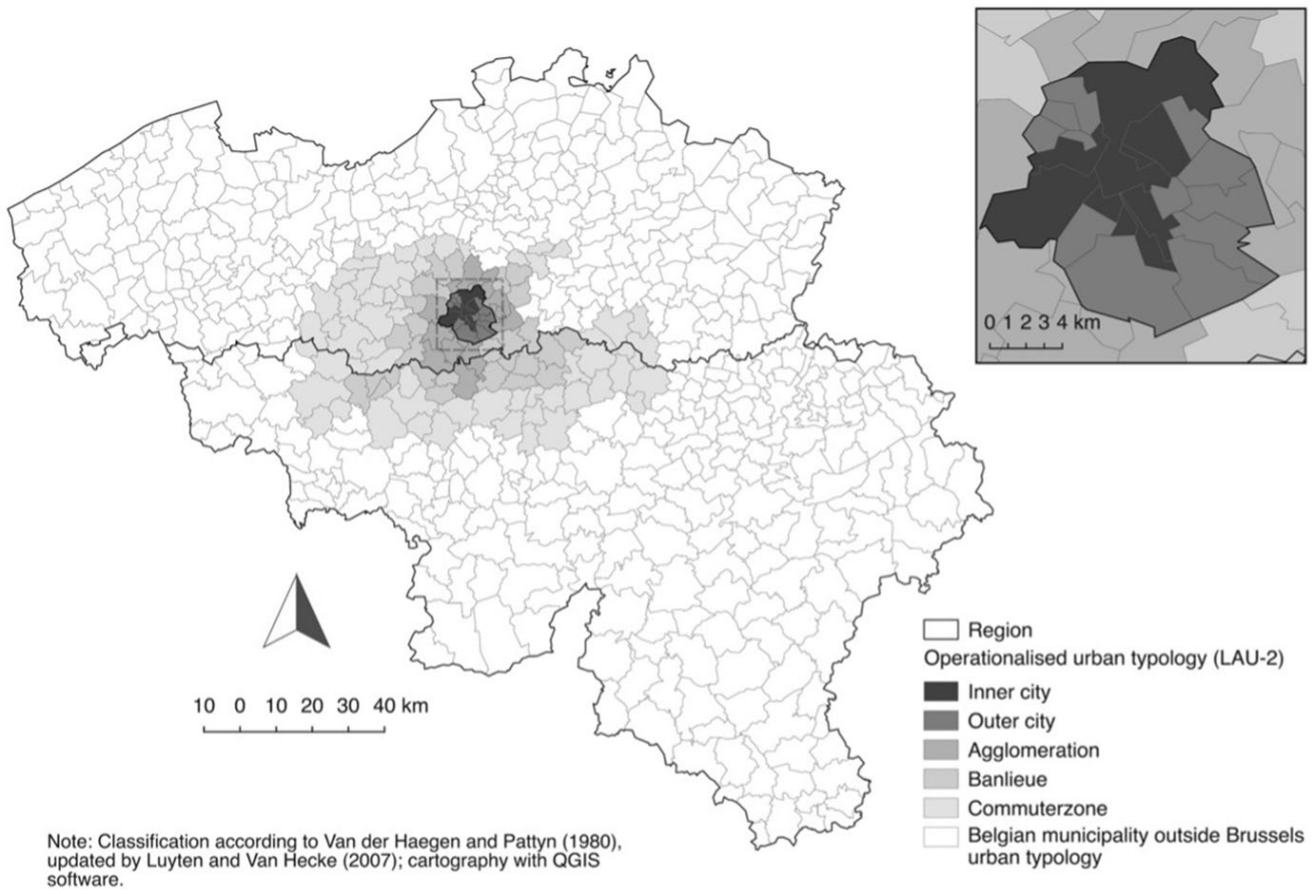
Classification and geography of the extended Brussels urban area

Controversial immigration debates, urban disturbances, and recent acts of terrorism have reignited concerns regarding the links between segregation, migration, citizenship, and national security in Belgium. These apprehensions have prompted policymakers to view the clustering of ethnic minorities, particularly Arab and Muslim people, in often economically disadvantaged areas as a potential threat to social integration and cohesion, relying on the assumption that individuals who reside in divided communities have limited interactions with people from diverse backgrounds. Hosting a wide range of countries of origin and migration motives and being one of the most diverse cities worldwide, Brussels is at the heart of these debates. The city shows marked geographic fractures between wealthier immigrants and immigrants who lack human or economic capital (at arrival) (Van Mol & de Valk, 2016). This spatial polarisation roughly coincides with the territorial expansion of nineteenth-century Brussels before World War 1 (inner-part) and the later process of urbanisation (outer-part), illustrated in Fig. 1. Despite variation at municipal and neighbourhood level (as detailed by Costa & de Valk, 2018b; Otavova et al., 2023), the most deprived and ethnically dense neighbourhoods are predominantly located within inner-city areas, particularly in the continuous zone around the historical city centre where more than 40% of working-age residents fall into the low-income bracket, and high-income earners are notably scarce—the so-called poor croissant^1^—, whereas more distinct concentrations of wealthy and native-born/White-European persons are found in the outer-city, where rental prices are generally higher than in the inner-city (Costa & de Valk, 2018a, 2018b; Imeraj et al., 2018a). Labour migrant descendants and newcomers from outside the ‘Walled World’^2^ have settled and continue to do so in the central former industrial nineteenth-century neighbourhoods, characterised by substandard dwellings in the private rental market—long inhabited by low-income households and left behind by the Belgian middle-class—and show limited signs of dispersal, unless to the adjacent neighbourhoods (Imeraj et al., 2021; Van Hamme et al., 2016). Wealthier (European) newcomers in contrast have mainly settled in more affluent neighbourhoods in the southeast and in the urban fringe. To a great extent, this is due to the (neo)liberal housing market, the lack of public dwellings (only 8% of the housing stock), the discriminatory practices and the ongoing gentrification, which steer migrant populations towards particular neighbourhoods and areas in the city (Dessouroux et al., 2016; Ghekiere & Verhaeghe, 2022; Imeraj et al., 2018a; Van Criekingen, 2006). Settlement in the own ethnic community may then become the dominant strategy to achieve residential satisfaction, cultural-specific resources compensating the lack of socioeconomic resources (Van der Laan Bouma-Doff, 2007). This dynamic may be further intensified as marginalised minority groups in Belgium face severe obstacles to social mobility over a lifespan and across generations, limiting opportunities to realise their residential preferences and consolidating spatial disparities (Phalet et al., 2007).

Since the early 1990s, Brussels’ authorities have prioritised targeted and multifaceted interventions in disadvantaged urban areas, particularly through Neighbourhood Contract programs (Sacco 2010, as cited in Van Hamme et al., 2016). These policies embrace a commitment to combat residential segregation by fostering more 'mixed communities’ as they are primarily based on the premise that spatial segregation exacerbates social inequalities through the so-called neighbourhood effects (Galster & Sharkey, 2017). Promoting greater social diversity in poor neighbourhoods is considered a strategy to mitigate potential detrimental neighbourhood effects. This has proven difficult, given the uneven distribution of population and housing prices/conditions in (and outside) Brussels which led to specific urbanisation dynamics and urban sprawl in its Flemish and Wallonian hinterland since the 1960s; processes are linked to migrant background and community resources, socioeconomic resources and intergenerational support, and the availability of affordable and/or social housing (Dessouroux et al., 2016). The specificity of the suburbanisation process—predominantly among the wealthiest individuals and families but increasingly among migrant (descendant) families too (de Valk & Willaert, 2012; Van Criekingen, 2009)—expands patterns of socioeconomic and ethnic concentration beyond the city’s boundaries into the Brussels agglomeration and further away (Fig. 1). At the macro-level, this is reflected in the distinct spatial separation between impoverished central neighbourhoods and prosperous outskirts, as illustrated by Costa and de Valk (2018b). The enduring spatial constraints that give rise to these conditions are deeply entrenched in the physical structure of the city, often in tangible ways. This includes elements like housing projects, main roads, and railways, as well as geographical features that are either permanent or change very slowly, such as bodies of water (the Canal zone from the Northeast to the Southwest) and designated natural zones (such as the Sonian forest in the Southeast).

As the European capital, Brussels provides an interesting empirical case to explore individual agency for understanding mechanisms that shape urban population geography since its cosmopolitan context bears important similarities with other cities, in Europe and beyond, but also distinct differences. Guided by the particular Brussels dynamics and geographies, we focus on movements that depart from poor migrant-dense concentration areas located in the Brussels inner-city to wealthier Brussels areas or suburban and further-away destinations outside Brussels.

## Data

This study used a longitudinal database covering the entire *de jure* population in Belgium between 1/10/1991 and 1/1/2006. Data consist of a double linkage at the individual level using a unique anonymised person identification number that enables linking (i) the 2001 Belgian Census to Register data on residential moves between 1/10/2001 and 1/1/2006; and (ii) the 2001 and 1991 Censuses, allowing to link individuals with their parents and providing a rich set of socio-demographic and socioeconomic variables to differentiate parental, ethnic and residential background prior to relocation. At the time of this research, a record linkage between more recent Censuses was unavailable. However, the unique qualities of the data still enable us to conduct in-depth and robust analyses. They provide valuable insights into the mechanisms driving (re)production of spatial patterns, which, in turn, support theoretical reflections.

Empirical analyses considered a 10-year cohort of 23- to 32-year-old Brussels inner-city residents at the time of the 2001 Census. For reasons of confidentiality, only inner-city neighbourhoods with more than 250 private households were considered. This downtown zone consists of seven municipalities: Anderlecht, Bruxelles, Ixelles, Molenbeek-Saint-Jean, Saint-Gilles, Saint-Josse-ten-Noode and Schaerbeek (Fig. 1). Respondents who moved abroad (*N* = 2,346) or who died (*N* = 198) during the follow-up period 2001–2006 were omitted. To determine achieved and parental education and migration background, analyses were based on individuals who were present in the 1991 census, who survived or did not emigrate during the post-1991 decade, and who lived with their parents at that time. To ensure that individuals completed their educational training in Belgium, first-generation migrants were excluded from the analysis. Respondents still enrolled in full-time education in 2001 (*N* = 1,838) were not included. The final study population consisted of 52,514 respondents.

## Measurement and Analytic Approach

The outcome variable, *residential mobility*, differentiated four mobility-categories and relied on a comparison of the municipality of legal residence—corresponding to Local Administrative Units (LAU) 2 level—on 1/10/2001 and 1/1/2006. Movers thus relocated across LAU-2 units; relocations within municipal boundaries were not captured. All moves depart from downtown Brussels; destinations were defined in line with the urban classification of Van der Haegen and Pattyn (1980) and Luyten and Van Hecke (2007) (Fig. 1). This delineation of urban centres and peripheries was developed specifically for the densely populated Belgian territory, using the continuity of the built fabric as the main delineation criterion. The inner-city is made up of the historical heart—the *pentagon*—, where political, economic and cultural activities are concentrated, and of neighbourhoods that have been urbanised mainly in the nineteenth century, characterised by private old rental dwellings of poor quality, a high population density, many single-person households and a young age structure. The outer-city comprises wealthier uptown areas urbanised mainly from the 1950s onwards, characterised by larger living spaces and higher rental prices, middle-class families and higher mean incomes. The suburbs consist of the urban agglomeration, the banlieue and the commuter zone, and extends the central city with an urban fringe that consists of continuous buildings (houses and public buildings), industrial and economic space, and parks. On average, Brussels suburbs have larger housing units and plots, and higher housing prices than the central city (Vastmans & Dreesen, 2021). While this area morphologically becomes somewhat less urban with increasing distance from the central city, its functionality is strongly oriented towards the city. As for areas beyond the Brussels suburban zone, regional cities generally boast higher costs compared to less urbanised or rural areas, featuring diverse housing inventories (Fednot; Vastmans & Dreesen, 2021). The outcome typology aims to capture the specific mobility dynamics that arise from the uneven Brussels geography and its broader context, and aligns with macro-scale patterns of socioeconomic fragmentation (Costa & de Valk, 2018b). The measure distinguished relocations (1) within the inner-city; and away from inner-city Brussels, (2) to outer-city areas in Brussels; (3) to Brussels suburbs^3^; or (4) to other destinations located in Belgium. Non-movers were the baseline.

The three main explanatory variables are as follows: *educational attainment* (Census 2001), *parental education* (Census 1991) and *ethnic background* (Census 1991 and 2001). The *educational attainment* of the young adult population was measured in 2001 and defined by the International Standard Classification of Education (ISCED). The measure distinguished four groups: no or primary education (ISCED 0–1), lower secondary education (ISCED 2), higher secondary and post-secondary education (ISCED 3–4) and tertiary education (ISCED 5–6). *Parental education* was based on both parents’ highest degree (measured in 1991) instead of fathers’ degree only because of the crucial role of mothers’ education in children’s achievement, the increase in female participation in higher education and labour and the rise of single-parent (-mother) households (Beller, 2009; Lampard, 2007). The indicator aligns with the *educational attainment* classification. Missing values constituted a rest group for both educational indicators and were included in the analysis as separate categories. *Ethnic background* was defined by individuals’ own and parental national background at the time of the 2001 Census. Children of foreign origin encompassed respondents meeting one of the following criteria in 2001: those with a non-Belgian nationality, those born with a non-Belgian nationality, and those born with Belgian nationality but with one or both parents having a non-Belgian nationality at birth. The indicator distinguished individuals of Belgian, North- and West-European, South-European, East-European, Turkish and Moroccan background and a remainder group mainly consisting of non-Western origins (95.0%). This classification effectively represents the primary migrant groups as it encapsulates the key stages of Belgian migration history.

Models included statistical control for a series of socio-demographic, socioeconomic and location-specific characteristics, all derived from the 1991 and 2001 Census data. Socio-demographic characteristics included *sex* (dummy), *age* in 2001 (age squared to control for nonlinear effects) and *household transition* prior to moving. The latter indicator distinguished six transitions from being a child in the parental household in 1991 to child (status quo), single, in union without children, in union with children, single parent and other household positions in 2001.

Socioeconomic indicators were measured in 2001 and included *employment status* and *housing tenure* as an approximation of financial prospects and constraints for future residential mobility. *Employment status* distinguished employed from unemployed individuals, including those who actively look for a job and non-active unemployed individuals. The *tenure*-variable distinguished owners from renters. Both indicators included a rest group consisting of respondents with unknown information for these socioeconomic variables which were included in the analysis as active missings.

Contextual indicators included neighbourhood *minority concentration* and *deprivation*, *co-ethnic resources, childhood residential context*, and *proportion affordable dwellings.* These covariates all relate to young inner-city residents’ neighbourhoods of residence in 2001 and aimed to integrate potential structural restrictions to residential relocations. Neighbourhoods are defined as statistical sectors, i.e. small geographical units with fixed boundaries (comparable with wards or census tracts). These sectors were delineated by Statistics Belgium according to structural social, economic, urban development or morphologic characteristics. *Minority concentration* was calculated as the share of residents with an ethnic minority background (cf. supra) in the total neighbourhood population; calculations being restricted to inner-Brussels neighbourhood populations registered in 2001. All neighbourhoods were ranked according to this percentage and divided in quintiles, assigning equal populations to each quintile. The first quintile represented the 20% residents living in the most minority dense inner-city neighbourhoods; the fifth quintile included the 20% residents living in the least minority dense inner-city neighbourhoods. Deprivation was measured by the Carstairs index of deprivation, adapted to the Belgian context (Deboosere et al., 2006). For each inner-city neighbourhood, the index considered the percentages of unemployed men aged 18–64, of households without a car and of low-educated residents aged 25–64 (i.e. primary education at most) present in 2001. The index was computed by summing *z*-scores by neighbourhood, weighted by the neighbourhood population size in 2001. Neighbourhoods were then ranked based on their final score and divided into quintiles, assigning equal population counts to each quintile. The first and the fifth quintile included the 20% residents living in the most and least deprived inner-city areas, respectively. Available *co-ethnic resources* were approximated by the location quotient (LQ), a group- and location-specific measure that captures the area-specific ethnic composition and geographic dispersion within cities (Brown & Chung, 2006). Our indicator differentiated the same groups as the ethnic background covariate. Here too statistical sectors acted as the basic spatial unit. The LQ was computed as the share of, for example, Turks in the neighbourhood population relative to the share of Turks in the entire central city. So, the LQ equals one in case the proportion of Turks in the neighbourhood is consistent with their proportion in Brussels overall; a value higher (lower) than one indicates that there are proportionally more (less) Turks in the neighbourhood than in the city. To control for differential duration of residence and return-migration to the parental home region, models also integrated *childhood residential context,* a measure that compares the residence in 2001 to that in 1991, irrespective of any household transition. Changes in residence were classified according to geographic proximity, separating four groups: still living in the same neighbourhood, lived in another neighbourhood in the same municipality, lived in another Brussels municipality, lived outside Brussels. The proportion of affordable dwellings was calculated as the share of private rental properties with monthly charges below €750 in the inner-city Brussels housing market in 2001; calculations were done at LAU-2 level. A quadratic term was included to control for nonlinearity.

We conducted multinomial logistic regression analyses to predict immobility as well as moves within the inner-city, to the outer-city, to suburbia or to further-away destinations as a function of individual, parental and contextual characteristics. Models were built through stepwise adding of covariates; interaction effects were included for educational attainment and parental education, for educational attainment and ethnic background, and for parental education and ethnic background. The assessment of the deviance allowed to test the fit of the model at each stage of model building. We only present the final full model; results of the sequential model building are available upon request.

## Results

### Young Adults’ Educational and Ethnic Background

To provide a picture of young adult profiles, Table 1 presents the educational attainment of young Brussels inner-city residents by parental education and ethnic background. Four out of five young adults have finished at least mandatory secondary education (compared to 46% among their parents). Cross-classification with parental education illustrates a significant positive association, proportions of highly educated young adults increasing with parental education. In line with Phalet and colleagues (2007), attainment patterns are ethnic-specific. Turkish and Moroccan respondents attain lower levels of education compared to their native-Belgian peers, whereas South- and East-European respondents take an intermediate position. The inferior educational performance of labour migrant descendants suggests a heritage of the educational distribution of the parent-generation despite educational progress.

**Table 1:** Educational attainment of young Brussels inner-city residents, by parental education and ethnic background, 2001

	Educational attainment (%)					
	No formal -primary	Lower secondary	Higher secondary	Tertiary	Unknown	Total
Parental education						
No formal—primary	5.9	24.3	34.8	21.3	13.8	19,251
Lower secondary	3.2	15.8	31.3	39.6	10.1	5,107
Higher secondary	1.7	7.9	23.4	59.1	7.9	9,370
Tertiary	0.7	2.9	12.2	77.2	7.0	10,219
Unknown	5.2	20.9	31.8	27.1	15.1	8,567
Ethnic background						
Belgian-native	2.6	10.3	21.7	56.4	9.0	25,287
West-European	2.7	10.8	22.6	52.0	11.9	2,899
South-European	4.6	18.8	31.4	34.4	10.9	5,531
East-European	4.0	18.6	31.5	33.7	12.1	1,236
Turkish	7.6	28.8	35.4	14.1	14.0	3,345
Moroccan/Maghrebian	5.1	23.9	36.8	20.1	14.1	12,369
(Non-)Western	2.3	11.1	25.1	44.2	17.2	1,847
Total population	1,974	8,310	14,455	21,869	5,906	52,514
	3.8	15.8	27.5	41.6	11.2	100.0

Chi-Square test statistics are significant at the 0.001-level

### Moving (out)? Educational and Ethnic Selectivity

Table 2 illustrates residential mobility patterns by own and parental education and ethnic background. Out of the 52,514 downtown Brussels residents in 2001, the majority (67.7%) still lived there in 2006. Of them, 13.1% moved to another inner-city municipality whereas 54.5% remained in the same municipality. In total, 32.3% moved out of the inner-city to outer-city areas (12.5%), to the Brussels agglomeration (6.3%), the banlieue (3.7%), the commuter zone (3.6%) or another Belgian municipality (6.2%). Relocation types vary significantly by educational and ethnic background. Not moving is more common in the lower educational strata than in the higher strata, whereas higher education stimulates residential mobility, particularly when directed towards the outer-city or suburbia. Parental education operates much in the same way as achieved education; young adult mobility seems to be pushed by any parental degree above primary education but those with relatively high-educated parents tend to favour outbound relocations. These exit-movers count proportionally more Belgian-natives compared to movers within inner-city Brussels or non-movers. West-, South- and East-European adults show a relatively equal distribution of residential outcomes, whereas most Turks do not move and Moroccans stay in the inner-city, whether moving or not. The latter groups appear least inclined to leave the Brussels capital.

**Table 2:** Residential mobility types by educational and ethnic background, 2001–2006

	Residential mobility type (%)										
	Non-movers		Inner-city movers		Outer-city movers		Suburban movers		Longer-distance movers		Total population N (%)
	% Column	% Row	% Column	% Row	% Column	% Row	% Column	% Row	% Column	% Row	
Educational attainment											
No formal–primary	4.7	67.8	3.3	11.6	2.4	7.9	2.2	8.1	2.9	4.7	1,974 (3.8)
Lower secondary	18.4	63.4	15.3	12.7	11.5	9.1	12.3	10.6	10.8	4.2	8,310 (15.8)
Higher secondary	29.8	59.1	25.4	12.1	24.0	10.9	26.5	13.1	21.2	4.8	14,455 (27.5)
Tertiary	36.1	47.2	41.0	12.9	50.3	15.1	51.1	16.8	53.8	8.0	21,869 (41.6)
Unknown	11.1	53.6	15.0	17.6	11.7	13.0	7.9	9.5	11.4	6.3	5,906 (11.2)
Parental education											
No formal–primary	42.3	62.8	36.3	13.0	29.0	9.9	26.8	10.0	25.4	4.3	19,251 (36.7)
Lower secondary	9.0	50.7	8.2	11.1	9.6	12.3	12.9	18.1	12.3	7.8	5,107 (9.7)
Higher secondary	14.8	45.1	17.1	12.6	20.2	14.1	24.5	18.8	26.9	9.4	9,370 (17.8)
Tertiary	15.5	43.4	20.4	13.8	26.4	17.0	24.5	17.2	27.2	8.7	10,219 (19.5)
Unknown	18.4	61.6	18.1	14.6	14.7	11.3	11.3	9.4	8.2	3.1	8,567 (16.3)
Ethnic background											
Belgian-native	41.0	46.4	44.1	12.1	54.4	14.1	65.4	18.5	69.3	8.9	25,287 (48.2)
West-European	4.9	48.3	5.7	13.6	7.7	17.4	5.8	14.4	5.7	6.4	2,899 (5.5)
South-European	10.9	56.4	10.2	12.7	9.7	11.5	11.1	14.4	8.4	5.0	5,531 (10.5)
East-European	2.6	60.2	2.0	11.3	2.5	13.2	2.0	11.4	1.5	4.0	1,236 (2.4)
Turkish	8.8	75.2	5.1	10.5	3.1	6.1	2.8	5.9	2.4	2.3	3,345 (6.4)
Moroccan/Maghrebian	28.7	66.4	28.2	15.7	17.8	9.4	10.1	5.9	9.8	2.6	12,369 (23.6)
(Non-)Western	3.2	49.5	4.6	17.2	4.8	17.2	2.8	11.0	2.9	5.2	1,847 (3.5)
Total population	28,631 (54.5)		6,902 (13.1)		6,556 (12.5)		3,319 (13.6)		3,263 (6.2)		52,514

Chi-Square test statistics are significant at the 0.001-level

### Explaining Variability in Residential Mobility Types

**Fig. 2:**
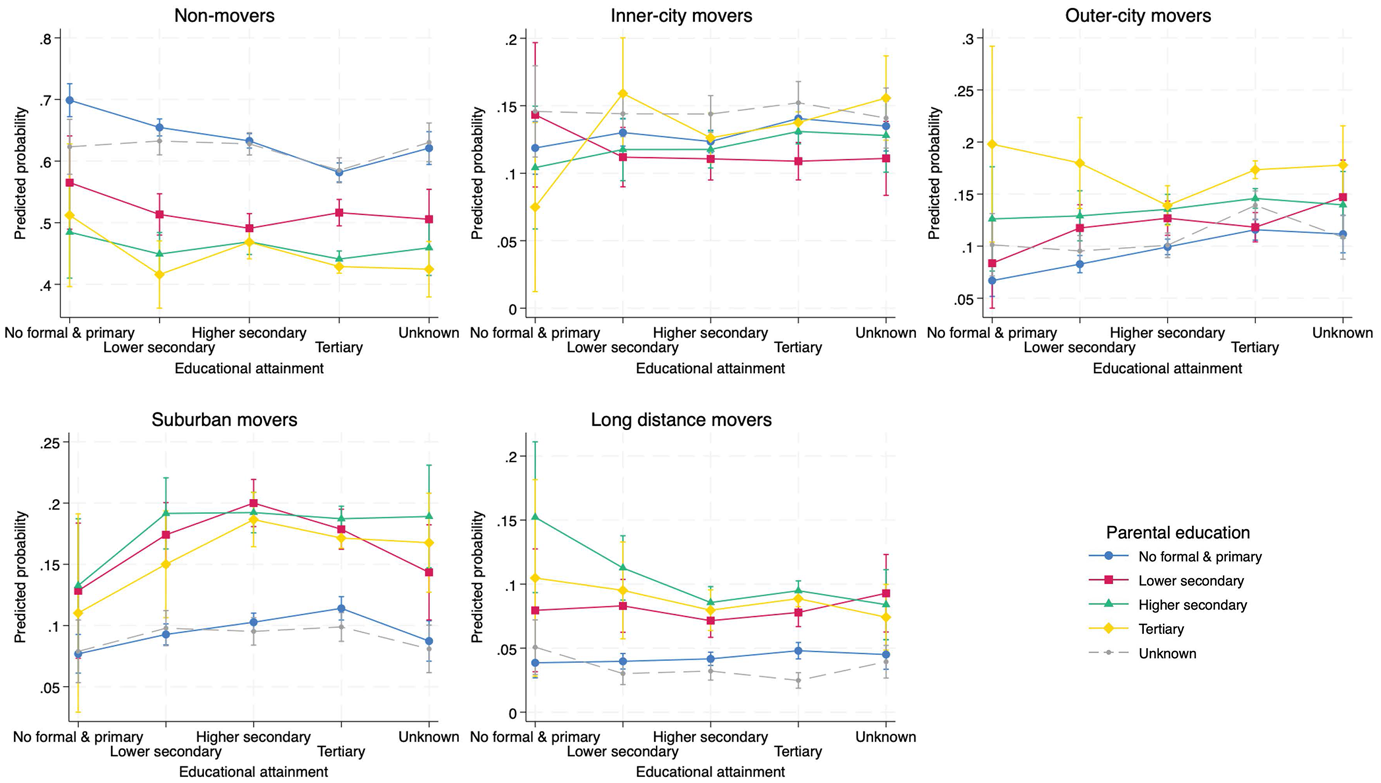
Interaction effect between educational attainment and parental education, adjusted predicted probabilities with 95% CIs for each residential mobility type from the extended multinomial model presented in Table 3, 2001–2006

**Fig. 3:**
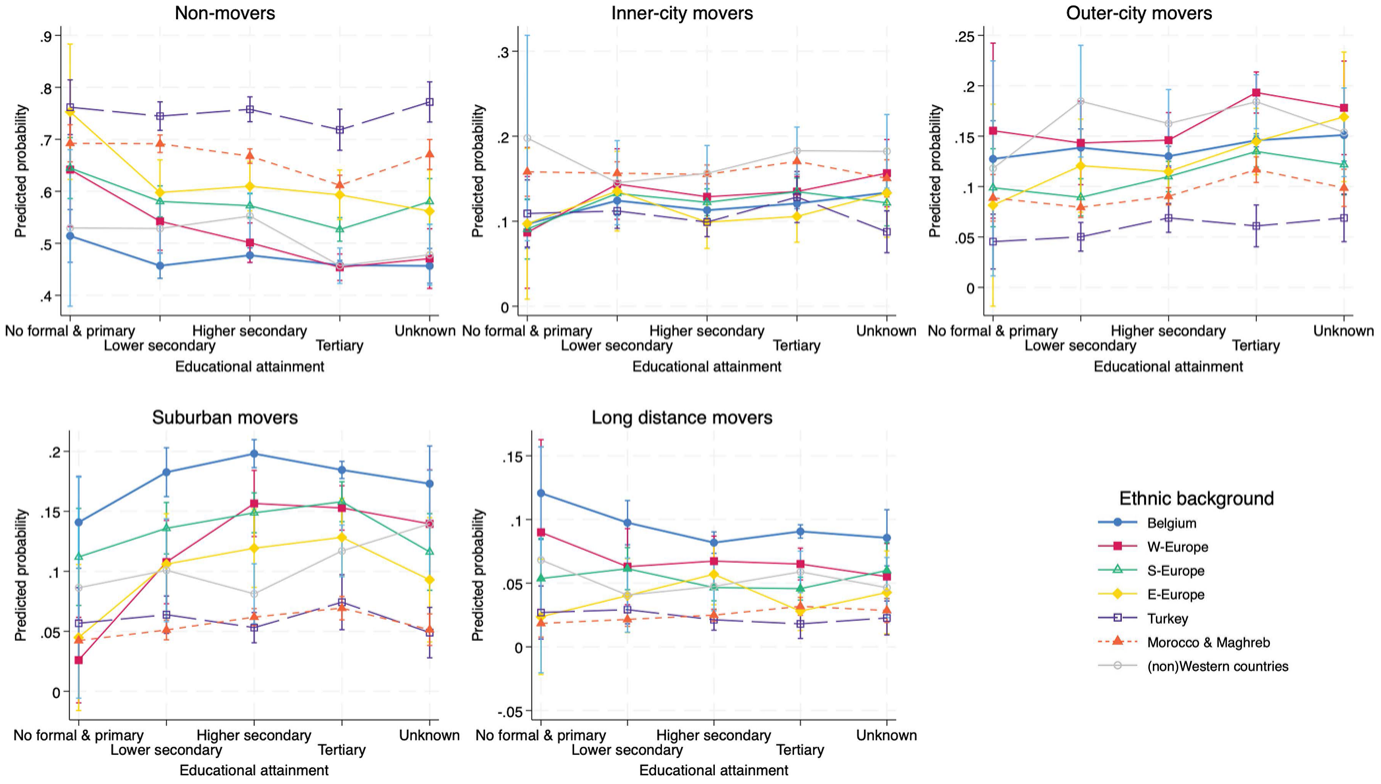
Interaction effect between educational attainment and ethnic background, adjusted predicted probabilities with 95% CIs for each residential mobility type from the extended multinomial model presented in Table 3, 2001–2006

**Fig. 4:**
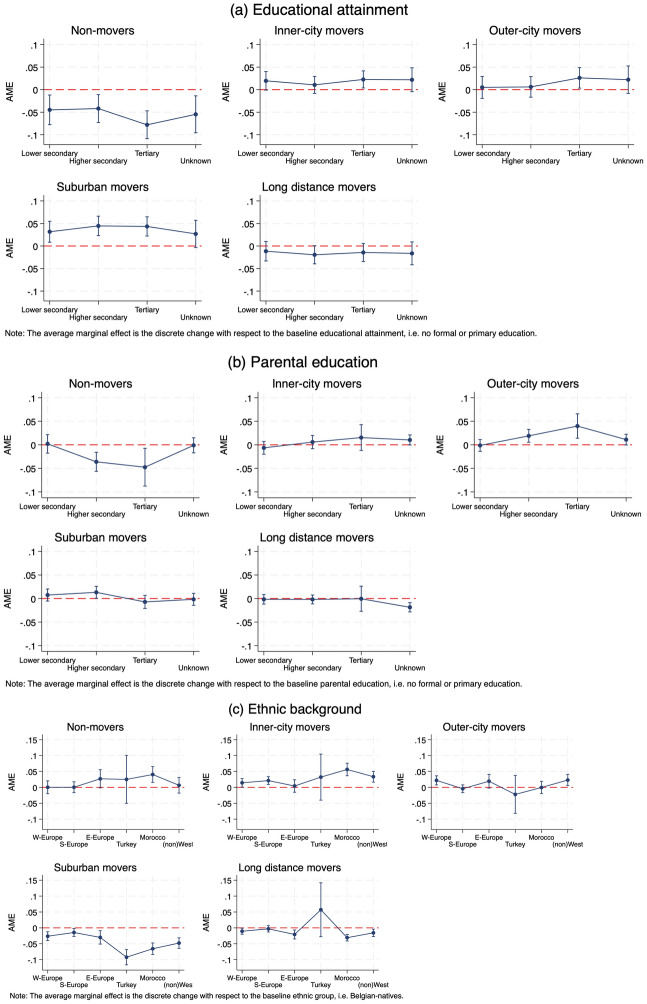
Average Marginal Effect (AME) of **a** educational attainment, **b** parental education and **c** ethnic background with 95% CIs on the probability of each residential mobility outcome from the extended multinomial model^a^, 2001–2006. ^a^Model includes educational attainment, parental education, ethnic background and interactions educational attainment*parental education, educational attainment*ethnic background and parental education*ethnic background, and socio-demographic, socioeconomic and neighbourhood characteristics; AME (dy/dx) for factor levels is the discrete change from the base level

As we have discerned from the above bivariate exploration of residential mobility types, there appears a significant degree of selectivity during adulthood mobility. To further investigate the role of education, we ran several baseline multinomial models (available in Appendix 2 and 3). The average adjusted predicted probabilities for residential mobility outcomes based on educational attainment, parental education and ethnic background in these baseline models, confirm this sorting of movers and stayers. In this section, we delve deeper into this selectivity and assess its persistence when accounting for additional background variables and considering potential interplays to understand if and how the impact of education is mediated by parental education and ethnic origin. Table 3 presents main effects of achieved education, parental education and ethnic background, as well as estimates of all control factors; Figs. 2 and 3 present full model interaction effects. To assess the significance of the effects, Fig. 4 shows the average marginal effects of educational and ethnic background in the full model. Frequencies of all included explanatory variables are provided in Appendix 1, separate for movers and non-movers.

**Table 3:** Adjusted predicted probabilities (Pr) for residential mobility types from the full multinomial model with interaction terms^a^, 2001–2006

	Non-Movers	Inner-city movers	Outer-city movers	Suburban movers	Longer-distance movers
	Pr [SE]	Pr [SE]	Pr [SE]	Pr [SE]	Pr [SE]
Achieved education					
No formal–Primary^#^	0.60 [0.015]	0.11 [0.009]	0.11 [0.011]	0.10 [0.011]	0.08 [0.010]
Lower Secondary	0.55 [0.007]	0.13 [0.005]	0.12 [0.005]	0.13 [0.006]	0.07 [0.005]
Higher secondary	0.56 [0.005]	0.13 [0.003]	0.12 [0.003]	0.14 [0.003]	0.06 [0.002]
Tertiary	0.52 [0.004]	0.14 [0.003]	0.14 [0.003]	0.14 [0.003]	0.06 [0.002]
Unknown	0.54 [0.014]	0.13 [0.009]	0.13 [0.010]	0.13 [0.011]	0.06 [0.008]
Parental education					
No formal—Primary^#^	0.55 [0.005]	0.12 [0.003]	0.12 [0.003]	0.13 [0.004]	0.07 [0.003]
Lower Secondary	0.56 [0.009]	0.12 [0.006]	0.11 [0.006]	0.14 [0.005]	0.07 [0.004]
Higher secondary	0.52 [0.009]	0.13 [0.006]	0.14 [0.006]	0.15 [0.005]	0.07 [0.004]
Tertiary	0.51 [0.020]	0.14 [0.014]	0.16 [0.013]	0.13 [0.006]	0.06 [0.013]
Unknown	0.55 [0.007]	0.13 [0.005]	0.13 [0.005]	0.13 [0.005]	0.05 [0.004]
Ethnic background					
Belgian-native^#^	0.53 [0.004]	0.11 [0.003]	0.13 [0.003]	0.16 [0.003]	0.07 [0.002]
West-European	0.53 [0.009]	0.13 [0.006]	0.15 [0.007]	0.13 [0.006]	0.06 [0.004]
South-European	0.53 [0.008]	0.13 [0.006]	0.12 [0.006]	0.14 [0.006]	0.07 [0.005]
East-European	0.56 [0.014]	0.12 [0.010]	0.15 [0.011]	0.13 [0.010]	0.05 [0.007]
Turkish	0.56 [0.038]	0.15 [0.037]	0.11 [0.030]	0.07 [0.012]	0.13 [0.043]
Moroccan/Maghrebian	0.57 [0.012]	0.17 [0.010]	0.13 [0.009]	0.09 [0.009]	0.04 [0.005]
(Non-)Western	0.54 [0.012]	0.15 [0.008]	0.15 [0.009]	0.11 [0.008]	0.05 [0.006]
Achieved education # Parental education^b^					
Achieved education # Ethnic background^b^					
Parental education # Ethnic background^b^					
Household transition					
Child–Child (stable)^#^	0.52 [0.006]	0.15 [0.005]	0.15 [0.005]	0.13 [0.004]	0.05 [0.003]
Child–Single	0.56 [0.004]	0.14 [0.003]	0.13 [0.003]	0.11 [0.002]	0.07 [0.002]
Child–Childless union	0.53 [0.005]	0.12 [0.003]	0.13 [0.003]	0.16 [0.004]	0.06 [0.002]
Child–Union with child(ren)	0.57 [0.005]	0.10 [0.003]	0.10 [0.003]	0.18 [0.004]	0.06 [0.003]
Child-Single parent	0.54 [0.010]	0.12 [0.007]	0.12 [0.007]	0.16 [0.008]	0.07 [0.006]
Child-other	0.51 [0.012]	0.16 [0.009]	0.14 [0.008]	0.12 [0.008]	0.07 [0.006]
Employment status					
Job^#^	0.53 [0.003]	0.13 [0.002]	0.13 [0.002]	0.15 [0.002]	0.06 [0.001]
No job	0.60 [0.005]	0.13 [0.003]	0.11 [0.003]	0.10 [0.003]	0.06 [0.003]
Unknown	0.54 [0.014]	0.15 [0.010]	0.12 [0.010]	0.12 [0.011]	0.07 [0.008]
Tenure					
owner^#^	0.65 [0.004]	0.09 [0.003]	0.09 [0.003]	0.12 [0.003]	0.05 [0.002]
Renter	0.50 [0.003]	0.15 [0.002]	0.14 [0.002]	0.15 [0.002]	0.07 [0.001]
Unknown	0.51 [0.007]	0.15 [0.005]	0.13 [0.005]	0.14 [0.005]	0.07 [0.004]
Age	0.54 [0.004]	0.13 [0.007]	0.12 [0.005]	0.13 [0.004]	0.07 [0.010]
Childhood residential context					
Same neighbourhood^#^	0.63 [0.005]	0.10 [0.003]	0.12 [0.004]	0.12 [0.004]	0.03 [0.002]
Same municipality	0.63 [0.005]	0.11 [0.003]	0.12 [0.004]	0.11 [0.003]	0.03 [0.002]
Same region	0.50 [0.004]	0.18 [0.003]	0.17 [0.003]	0.12 [0.003]	0.03 [0.001]
Changed region	0.47 [0.005]	0.14 [0.003]	0.10 [0.002]	0.17 [0.003]	0.12 [0.003]
Neighbourhood deprivation					
Q1–Least deprived^#^	0.49 [0.006]	0.15 [0.005]	0.15 [0.004]	0.14 [0.004]	0.06 [0.002]
Q2	0.52 [0.006]	0.14 [0.005]	0.13 [0.004]	0.14 [0.004]	0.06 [0.003]
Q3	0.55 [0.005]	0.14 [0.004]	0.12 [0.003]	0.13 [0.003]	0.06 [0.002]
Q4	0.57 [0.005]	0.11 [0.003]	0.11 [0.003]	0.14 [0.004]	0.07 [0.003]
Q5–Most deprived	0.59 [0.007]	0.12 [0.004]	0.10 [0.005]	0.12 [0.006]	0.06 [0.004]
Neighbourhood minority concentration					
Q1–Least concentrated^#^	0.58 [0.006]	0.09 [0.004]	0.10 [0.004]	0.16 [0.004]	0.06 [0.003]
Q2	0.57 [0.005]	0.11 [0.003]	0.13 [0.003]	0.14 [0.003]	0.06 [0.002]
Q3	0.56 [0.005]	0.12 [0.003]	0.13 [0.003]	0.13 [0.004]	0.06 [0.002]
Q4	0.53 [0.006]	0.16 [0.005]	0.13 [0.004]	0.12 [0.004]	0.06 [0.003]
Q5–Most concentrated	0.49 [0.008]	0.18 [0.007]	0.12 [0.006]	0.13 [0.006]	0.07 [0.005]
Location Quotient					
Q1–LQ < 0.73	0.52 [0.005]	0.14 [0.004]	0.14 [0.004]	0.14 [0.004]	0.07 [0.003]
Q2–0.73 < LQ < 1.08	0.52 [0.005]	0.16 [0.004]	0.13 [0.003]	0.13 [0.003]	0.06 [0.002]
Q3–1.08 < LQ < 1.40^#^	0.54 [0.005]	0.13 [0.004]	0.12 [0.003]	0.15 [0.004]	0.07 [0.003]
Q4–1.40 < LQ < 2.27	0.56 [0.005]	0.13 [0.004]	0.11 [0.004]	0.15 [0.004]	0.06 [0.003]
Q5–2.27 < LQ	0.60 [0.008]	0.11 [0.004]	0.13 [0.006]	0.11 [0.006]	0.05 [0.004]
Affordable dwellings (%)	0.52 [0.011]	0.16 [0.012]	0.13 [0.009]	0.13 [0.005]	0.06 [0.004]
AIC		126,674.3			
BIC		130,469.4			
-2 Log Likelihood		125,818.292			
Chi-Square		10,634.63	(df = 428)***		
Pseudo R^2^		0.0779			

^a^Model includes educational attainment, parental education, ethnic background and interactions educational attainment*parental education, educational attainment*ethnic background and parental education*ethnic background, and socio-demographic, socioeconomic and neighbourhood characteristics; ^b^ Interaction terms are visualised in Figs. 2 and 3; ^#^ Reference category in multinomial logit model

Table 3 illustrates how higher achieved education pushes the probability of residential mobility within the inner-city and to the outer-city or the suburbs, whereas longer-distance moves appear refrained as achieved education gets higher. For parental education, this pattern is observed as well, be it slightly more fragmented. Plotted interaction effects in Fig. 2 illustrate however that higher educational attainment initiates the best chances for spatial integration when parents have a degree higher than primary schooling. The probability to relocate is generally lowest among respondents with low-educated parents regardless of their own educational status. Spatial integration here mainly refers to outbound relocations towards Brussels suburbs. In contrast, young adults climbing from lowest educational strata do translate their educational upgrading into spatial moves towards the outer-BCR. In any of the above situations, parental education is of crucial importance up and beyond individual educational success. The gap in residential outcomes by parental education is largest for those who relocate to suburbia and for non-movers.

Focussing on ethnic heterogeneity and controlled for the broad set of variables, non-Europeans are more likely to relocate within inner-city boundaries, whereas suburban relocations remain most likely among native-Belgians and Europeans. Plotted interaction effects in Fig. 3 show a negative association between residential immobility and education in all ethnic groups. Non-movement is most probable for Turks, Moroccans and East-Europeans, regardless of their educational attainment. In contrast, residential moves out of Brussels are most likely for Belgian-natives at all educational levels. While increasing educational attainment favours ethnic minority community members to leave Brussels too, the beneficial effect of higher education appears lower compared to their native peers, especially for Turks and Moroccans.

To assess the substantive and practical significance of the estimated effects of educational attainment, parental education and ethnic background, average marginal effects (AMEs) were computed. AMEs in Fig. 4 express the average effect of a covariate on a particular outcome category; all AMEs with 95% CIs are provided in Appendix 4. In practice for example, an AME equal to −0.04 tells us that an otherwise ‘average’ young adult (i.e. having the mean value for all other variables included in the final model) who obtained lower secondary education is 4 per cent points less likely to be residentially immobile than similar ‘average’ individuals with primary education. In sum, AMEs for educational attainment illustrate that primary educated young adults are significantly more likely to stay put and less likely to move to the Brussels suburbs, all else equal. While inner- and outer-city movers seem to benefit from higher education too, the average marginal effect is not significant. Regarding parental education, we expect a significant decrease in the probability of not moving for young adults with intermediate and high-educated parents compared to their peers with low-educated parents. The increase in the marginal effect of parental education is visible—but not significant—among all other movers, except suburban movers with tertiary educated parents. Everything else kept equal, all minority groups are significantly expected less likely to relocate to the suburbs than native-Belgians. Most marked and significant AME-differences are observed between native-Belgians and non-European young adults.

Table 3 additionally shows that socioeconomic and socio-demographic indicators have a distinct role and play out as expected. Suburbanisation is a phenomenon that is associated with lifecycle components, such as family formation and childbearing, housing situation and having a (stable) job that provides sufficient financial resources. Long-term childhood exposure generates residential immobility. Location-specific factors generally reveal that neighbourhood context is projected onto inner-city adults’ residential paths. Neighbourhood deprivation is markedly associated with a gradually increasing likelihood of residential immobility and, more moderately, longer-distance moves. In contrast, deprivation is negatively associated with relocations in the inner-city, the outer-city and, less pronounced, the Brussels suburbs. Residence in minority concentration areas gradually stimulates residential moves within downtown Brussels and pushes mobility towards uptown Brussels or further-away destinations, while it reduces suburban relocations. Suburban and longer-distance moves are least likely for those living in pronounced co-ethnic communities. In general, the more spatially concentrated a community, the less likely a young member moves. Compared to the 20% neighbourhoods with moderate co-ethnic representation, the absence of a co-ethnic community alternatively pushes relocations within the inner-city or towards the outer-city.

## Discussion and Conclusion

This paper expands ongoing research efforts in segregation studies by paying closer attention to the subtle ways in which factors and mechanisms in traditional spatial assimilation theory affect residential behaviour. To do so, we took an explicit intergenerational and multi-ethnic perspective, investigating whether and how similar educational attainment forges differential residential moves depending on social and ethnic origin. Although traditional ideas of socio-spatial integration generally hold, our approach shows that educational success begets residential moves away from the inner-city predominantly among young adults from Belgian-native or well-educated families. Throwing off the influence of ‘background’ in trying to acquire suitable housing in a nice (suburban) living environment seems hardest for those starting at the bottom echelons, particularly in non-western minority populations.

Overall, education stimulates mobility. Yet, educational progress of labour migrant descendants prompts residential moves within the constrained areas of the inner-city only. Especially Turks and Moroccans appear to end up in similar downtown areas as the ones they come from, if they move at all. The subpar attainment of educational credentials compared to their Belgian counterparts (Phalet et al., 2007) is projected far into their professional careers, which, in turn, cuts back financial returns to education and renders fewer qualitative dwellings affordable (Lindley & Machin, 2012). Labour migrant parents, having fewer resources to pass on, also impede their children’s residential trajectories (Hostenbach, 2018) (see Appendix 5). This underscores how the wealth background of parents has significant spatial implications, contributing to the reinforcement of existing socio-spatial disparities and the creation of novel ones over generations (Bailey et al., 2017; Gustafsson et al., 2017; Hermansen et al., 2022; Hostenbach, 2018; Pais, 2017, 2021). Living most segregated, Moroccan and Turkish clustering prevents dispersal yet may foster solidarity more than any other ethnic community (Peach, 1996). The availability of human and social capital within the local community may act as an important counterbalance to (fewer opportunities to get higher) educational attainment for individuals living in resource-scarce households (Galster & Sharkey, 2017); the decision to stay in the same area could reflect a (voluntary) coping strategy (Patacchini & Zenou, 2011). The increase in owner-occupation among Turks and Moroccans moreover makes the settlement areas of these communities very durable (Kesteloot & Cortie, 1998) and provides a potential alternative ethnic segment in the housing market. This form of ethnic support and investment in inner-city housing may have far-reaching consequences for spatial segregation in urban areas. In contrast, relocations within downtown Brussels could be involuntary because this particular group is prevented to relocate elsewhere by external factors, such as affordability of housing in uptown or suburban areas, or discrimination in the housing market. Evidence from field experiments and correspondence testing in the labour and housing market has shown discrimination against individuals with minority-sounding names (especially Muslim/Arab) in Europe (Flage, 2018; Quillian et al., 2019) and Belgium (Ghekiere & Verhaeghe, 2022; Lippens et al., 2022). Following the domains (van Ham & Tammaru, 2016) or multiple-context approach (Park & Kwan, 2017), discrimination against ethnic minorities in the job market is expected to decrease the economic resources available to immigrants, thereby restricting their ability to afford housing in affluent areas with high property prices. While it was beyond the scope of this paper to pinpoint down the exact role of these factors, discriminatory practices and gentrification increasingly mortgage access to affordable qualitative dwellings (Ghekiere & Verhaeghe, 2022; Van Criekingen, 2009). Moreover, when intergenerational persistence in context is substantial, this means that most adults living in affluent neighbourhoods grew up in similar areas, and the same applies to those residing in less-privileged neighbourhoods, even more so for minoritised population groups (Hermansen et al., 2022; Pais, 2017). This segregation results in a reduced likelihood of early-life interactions with individuals from diverse social or ethnic backgrounds, potentially leading to a decreased sense of affinity and trust towards those who differ from oneself and fewer opportunities, particularly if this persistence is not a matter of choice but rather a result of limited residential options (Galster & Sharkey, 2017; Gustafsson et al., 2017). Of course, spatial assimilation could actually happen at a lower geographic level than the municipal radius. The register data do not allow to assess movements at the neighbourhood level and cannot provide insights into the (lack of) improvement of neighbourhood conditions that go hand in hand with an inner-city relocation. This is the most important limitation of this study. However, because levels and patterns of segregation of these minority populations in Brussels are consistent at any geographic scale, spatial assimilation at neighbourhood level may extend to socio-spatial paths at LAU-2 level (Imeraj et al., 2018a). Moreover, it has been shown that acquaintance with the locality refrains non-Western migrants from leaving Belgian metropoles, even when having obtained a university degree (Imeraj et al., 2018b). Exploring socio-spatial integration by ethnicity at various geographic scales while considering an elaborate set of local structural characteristics would be a valuable extension to our work.

BCR-leavers tend to consist of a selective native-Belgian and well-educated group of young adults, which augments further minority concentration and relative deprivation in inner-city Brussels. A (temporal) residence in downtown Brussels supplies young native-Belgian adults—with cultural rather than financial capital—with living conditions that ‘are particularly suited to the specific social reproduction needs […] in both familial and professional transitional positions’ (Van Criekingen & Decroly, 2003, p. 2455), but does not halt suburbanisation in a later life stage. Overall, this may reflect a moving behaviour aimed at educational homogeneity (Musterd et al., 2016; van Gent et al., 2019). Importantly, given this native selection-mechanism persists in all educational groups, it could be the result of the decreasing affordability of the Brussels rental market as well (De Laet, 2018; Van Criekingen, 2006), which can be expected to have aggravated in the decades following the observation period as a consequence of the massive rise in housing prices in Brussels, the relatively few public dwellings (around 40,000 units) and the ongoing housing crisis.^4^ While today’s suburban housing prices are still among the highest, larger houses in more sparsely populated Brussels suburbs may offer greater value for the same price. Suburbanisation then potentially (and increasingly) manifests a (relative) spatial downgrading, either resigning to substandard housing in order to diminish the gap between income and housing costs, given the absence of public housing opportunities, or willing to exchange the city and its amenities in favour of larger and greener spaces (for the same cost) (Dessouroux et al., 2016). In contrast, recent data from the Brussels Institute for Statistical Analysis (BISA) indicate that the incomes of departing residents of Brussels are generally higher than those of individuals who remain in the region (and newcomers). This dichotomy potentially is even more pronounced for more distant destinations, as the economic disparity between polycentric metropolitan Flanders and Wallonia has led to a more significant increase in prices in regional cities than in peripheral or rural areas, creating substantial variation within this socio-spatial category (Vastmans & Dreesen, 2021). While these relocations accounted for only 6% of moves among young adults residing in inner-city Brussels, future research should further uncover who is pushed out of the city and why.

While neighbourhood context was not at the heart of this study, our analyses show decisively that poor neighbourhood conditions impede any relocation and minority concentrations deter suburban settlement, beyond individual features, thereby highlighting that spatial opportunity structures are important (Galster & Sharkey, 2017). Although minority dense and disadvantaged areas are often viewed as problematic and their reputation tends to lead to residential dissatisfaction and the intention to flee such neighbourhoods, particularly among white/native people (Permentier et al., 2009; van Ham & Clark, 2009), Brussels young adults do not (or are not able to) translate residence in poor minority dense neighbourhoods into leaving the inner-city in favour of suburbia. Moves within the Brussels capital, however, appear to be stimulated by increasing minority concentration and by co-ethnic scarcity. This suggests that young adults prefer to live among co-ethnics but less so among other ethnic group populations. Testing whether or not this is true for all educational achievements and parental legacies would be an interesting venue for future research.

In other words, while we found partial evidence for traditional mechanisms of socio-spatial integration, there is a deep-rooted selectivity within the socio-spatial sorting process: high education generates greater opportunities to escape downtown Brussels for individuals with a Belgian or well-off background than for individuals with low-educated or non-European parents, in particular when oriented to suburbia. To draw firm conclusions about this native-migrant discrepancy in leaving the urban area, the role of socioeconomic resources facilitating residential mobility should be further disentangled from the impact of (diverging) social norms and values with regards to residential aspirations. In sum, our results call for more comparative studies that contrast urban locations with a distinct housing market, institutional framework and migrant stock as a way ahead to understand migrant-specific pathways to socio-spatial integration.

Our study had several limitations. First, educational background is difficult to interpret, education being a relative measure within the societies that deliver educational training and degrees. Sensitivity analysis based on alternative classifications of education^5^ produced analogous estimates however. Secondly, missing values need caution as cases with incomplete information appear not random. Sensitivity analyses provided adequately similar estimates when omitting respondents with missing educational and socioeconomic information. Thirdly, this paper has exclusively focussed on education, leaving occupation and income aside. While this is mainly prompted by inaccurate information in the Belgian census, it makes sense to focus on education, being the main mechanism through which (dis)advantages and social status are transmitted from parents to children (Johnson et al., 2010). Furthermore, education is a relatively stable indicator of socioeconomic position compared to occupational status, especially among young adults (Bailey & Livingston, 2008). Admittedly also, the variations within the (sub)urban types are not captured by the current residential delineation and categorisation, nor could we account for potential changes over time due to, for example, gentrification. Hence, the use of a different urban typology may result in more nuanced results. Still, our analyses yield insight into socio-spatial integration trends during the study period and provides an overall picture for this specific Brussels geographic typology.

In conclusion, this empirical study on Brussels focussed on spatial integration as a function of educational attainment, contrasting residential trajectories by parental educational resources and ethnic background. The analysis showed how inherited opportunities and barriers to social and residential movement perpetuate socio-spatial inequalities in ethnic minority and majority populations. While the observed residential mobility patterns pointed at the existence of complex sorting mechanisms within and out of the area, it remains to be answered why some do not profit from an educational gain to the same extent as others, leaving still a considerable scope for future research within and beyond this case study area. With this, our study offers some new analytical entry points into socio-spatial integration research that align with the recent call for an intergenerational, multi-ethnic and spatial approach to more fully understand the salient role of education in divergent residential behaviour and residential segregation.

## Supplementary Information

Below is the link to the electronic supplementary material.

Supplementary file1 (PDF 4599 KB)

## Data Availability

The data that support the findings of this study are available from Statistics Belgium but restrictions apply to the availability of these data, which were used under license for the current study, and so are not publicly available.

## Footnotes

1 Poor areas also known as the “poor croissant” encompass parts of the centre, Schaarbeek, Saint-Josse, Molenbeek, and Anderlecht; the crescent reflects the uneven spatial distribution of social inequalities and housing/rental prices across the Brussels territory.

2 Recently, there has been a proposal to adopt the term 'Walled World,' highlighting how 14% of the global population resides behind barriers or walls, restricting entry based on wealth. This is evident in physical walls, like those in the Palestinian Occupied Territories and Trump's 'border wall' between Mexico and the USA. Additionally, it symbolises political barriers, such as 'Fortress Europe,' aimed at preventing migrants and refugees from entering the continent. Of course, the classification of countries and people reveals a complex problem with no straightforward solution, inviting diverse perspectives. Recognising the context-dependence of terms is crucial. While certain terms may be fitting in specific contexts, movements, or political landscapes, they may not universally apply. Merely proposing alternatives might not address the issue, as new terms could also risk exclusion or marginalisation (Kahn, 2022).

3 A more detailed residential categorisation was applied in earlier stages of model building, separating the agglomeration, the banlieue and the commuter zone. Because parameter estimates were highly similar, residential outcomes were grouped together in one category *‘Brussels suburbs’*.

4 Property prices have risen continuously at a high pace so that they have more than doubled over the last 20 years according to the Financial Stability Report 2020 (p.110) of the National Bank of Belgium; averaging 556,000€ for a single-family house and 285,000€ for an apartment. 90% of the rental properties concern apartments (Conferderatie van Immobilliënberoepen Vlaanderen (CIB)) and the average monthly rental cost has reached 1,188€ in 2023 but hides huge geographic variation, with most expensive accommodations in Elsene and Sint-Pieters-Woluwe and ‘cheapest’ in Anderlecht, Laken and Koekelberg according to the Federatie van het Notariaat (FedNot).

5 11 categories (no formal, primary, lower secondary vocational, lower secondary technical, lower secondary general, higher secondary vocational, higher secondary technical, higher secondary general, higher post-secondary, tertiary non-academic, tertiary academic); five categories (no formal/primary, lower secondary, higher secondary, tertiary non-academic, tertiary academic); three categories (low, intermediate, high education).
