# Supplementary material for: Trapped in Place? Ethnic and Educational Heterogeneity in Residential Mobility and Integration of Young Adults in Brussels
Source: Eur J Popul. 2024 Jan 25;40(1):5. doi: 10.1007/s10680-023-09690-3 (PMC10811305; doi:10.1007/s10680-023-09690-3)

Appendix 1 Explanatory variables: Counts and proportions by movers and non-movers

	Non-movers		Movers	
	N	%	N	%
**Educational attainment**				
No formal - primary	1,338	4.7	636	2.7
Lower secondary	5,267	18.4	3,043	12.7
Higher secondary	8,538	29.8	5,917	24.8
Tertiary	10,322	36.1	11,547	48.3
Unknown	3,166	11.1	2,740	11.5
**Parental education**				
No formal - primary	12,098	42.3	7,153	30.0
Lower secondary	2,591	9.0	2,516	10.5
Higher secondary	4,229	14.8	5,141	21.5
Tertiary	4,434	15.5	5,785	24.2
Unknown	5,279	18.4	3,288	13.8
**Ethnic group**				
Belgian-native	11,731	41.0	13,556	56.8
West-European	1,399	4.9	1,500	6.3
South-European	3,119	10.9	2,412	10.1
East-European	744	2.6	492	2.1
Turkish	2,514	8.8	831	3.5
Moroccan/Maghrebian	8,210	28.7	4,159	17.4
(Non-)Western	914	3.2	933	3.9
**Employment status**				
Job	18,129	63.3	17,171	71.9
No job	7,641	26.7	4,171	17.5
Unknown	2,861	10.0	2,541	10.6
**Tenure**				
Owner	10,483	36.6	5,013	21.0
Renter	14,317	50.0	15,237	63.8
Unknown	3,831	13.4	3,633	15.2
**Household transition**				
Child - Child	7,899	27.6	4,286	17.9
Child - Single	7,838	27.4	8,637	36.2
Child - Childless union	4,103	14.3	5,047	21.1
Child - Union with child(ren)	6,817	23.8	4,102	17.2
Child - Single parent	1,245	4.3	940	3.9
Child - Other	729	2.5	871	3.6
**Residential exposure**				
Same neighbourhood	8,650	30.2	4,055	17.0
Same municipality	6,532	22.8	3,175	13.3
Same region	7,067	24.7	7,249	30.4
Changed region	6,382	22.3	9,404	39.4
**Neighbourhood deprivation**				
Q1 - Least deprived	4,604	16.1	5,765	24.1
Q2	4,383	15.3	4,299	18.0
Q3	5,070	17.7	4,748	19.9
Q4	8,237	28.8	5,486	23.0
Q5 - Most deprived	6,337	22.1	3,585	15.0
**Neighbourhood minority concentration**				
Q1 - Least concentrated	5,032	17.6	4,205	17.6
Q2	5,671	19.8	5,983	25.1
Q3	5,684	19.9	5,725	24.0
Q4	5,769	20.1	4,313	18.1
Q5 - Most concentrated	6,475	22.6	3,657	15.3

Location Quotient				
Q1 - LQ < 0.73	4,891	17.1	5,088	21.3
Q2 - 0.73 < LQ < 1.08	6,474	22.6	7,395	31.0
Q3 - 1.08 < LQ < 1.40	5,269	18.4	5,077	21.3
Q4 - 1.40 < LQ < 2.27	5,119	17.9	3,385	14.2
Q5 - 2.27 < LQ	6,831	23.9	2,894	12.1
	Mean	SE	Mean	SE
Age	27.5	0.016	27.2	0.017
Affordable rental dwellings (%)	37.9	0.024	38.4	0.028
Total	28,631		23,883	

Appendix 2

Table A2.1 Predicted Probabilities (Pr) for the residential mobility types from the baseline multinomial model[a], 2001-2006

	Non-movers	Inner-city movers	Outer-city movers	Suburban movers	Longer-distance movers
	Pr [SE]	Pr [SE]	Pr [SE]	Pr [SE]	Pr [SE]
**Achieved education**					
No formal – Primary[#]	0.68 [0.011]	0.12 [0.007]	0.08 [0.006]	0.08 [0.006]	0.05 [0.005]
Lower Secondary	0.63 [0.005]	0.13 [0.004]	0.09 [0.003]	0.11 [0.003]	0.04 [0.002]
Higher secondary	0.59 [0.004]	0.12 [0.003]	0.11 [0.003]	0.13 [0.003]	0.05 [0.002]
Tertiary	0.47 [0.003]	0.13 [0.002]	0.15 [0.002]	0.17 [0.003]	0.08 [0.002]
Unknown	0.53 [0.006]	0.18 [0.005]	0.13 [0.004]	0.10 [0.004]	0.06 [0.003]
AIC	135353.8				
BIC	135531.2				
-2 Log Likelihood	135313.822				
Chi-Square	1384.49	(df=16)***			
Pseudo R^2	0.0101				

Note: [a] Model includes educational attainment; [#] Reference category

Table A2.2 Adjusted predicted Probabilities (Pr) for the residential mobility types from the multinomial model[a], 2001-2006

	Non-Movers	Inner-city movers	Outer-city movers	Suburban movers	Longer-distance movers
	Pr [SE]	Pr [SE]	Pr [SE]	Pr [SE]	Pr [SE]
**Achieved education**					
No formal – Primary[#]	0.64 [0.011]	0.12 [0.007]	0.09 [0.007]	0.09 [0.007]	0.06 [0.006]
Lower Secondary	0.60 [0.006]	0.13 [0.004]	0.10 [0.004]	0.12 [0.004]	0.05 [0.003]
Higher secondary	0.57 [0.004]	0.12 [0.003]	0.11 [0.003]	0.14 [0.003]	0.05 [0.002]
Tertiary	0.51 [0.004]	0.13 [0.002]	0.14 [0.002]	0.15 [0.002]	0.07 [0.002]
Unknown	0.52 [0.006]	0.18 [0.005]	0.14 [0.005]	0.10 [0.004]	0.07 [0.003]
**Parental education**					
No formal – Primary[#]	0.61 [0.004]	0.13 [0.003]	0.10 [0.002]	0.10 [0.002]	0.05 [0.002]
Lower Secondary	0.51 [0.007]	0.11 [0.004]	0.12 [0.005]	0.18 [0.005]	0.08 [0.004]
Higher secondary	0.47 [0.005]	0.13 [0.004]	0.14 [0.004]	0.18 [0.004]	0.09 [0.003]
Tertiary	0.46 [0.005]	0.14 [0.004]	0.16 [0.004]	0.16 [0.004]	0.08 [0.003]
Unknown	0.61 [0.005]	0.14 [0.004]	0.12 [0.004]	0.10 [0.003]	0.03 [0.002]
AIC	134036.9				
BIC	134356.2				
-2 Log Likelihood	133964.908				
Chi-Square	2733.41	(df=32)***			
Pseudo R^2	0.0200				

Note: [a] Model includes educational attainment and parental education; [#] Reference category

Table A3.3 Predicted Probabilities (Pr) for the residential mobility types from the multinomial model[a], 2001-2006

	Non-movers	Inner-city movers	Inner – Outer	Inner – Suburbs	Inner – Elsewhere
	Pr [SE]	Pr [SE]	Pr [SE]	Pr [SE]	Pr [SE]
**Achieved education**					
No formal - primary[#]	0.64 [0.011]	0.12 [0.007]	0.09 [0.007]	0.10 [0.007]	0.06 [0.006]
Lower Secondary	0.59 [0.006]	0.13 [0.004]	0.10 [0.004]	0.13 [0.004]	0.05 [0.003]
Higher secondary	0.57 [0.004]	0.12 [0.003]	0.12 [0.003]	0.14 [0.003]	0.05 [0.002]
Tertiary	0.52 [0.004]	0.13 [0.002]	0.14 [0.002]	0.15 [0.003]	0.07 [0.002]
Unknown	0.52 [0.006]	0.17 [0.005]	0.14 [0.005]	0.10 [0.004]	0.07 [0.004]

**Parental education**					
No formal - primary[#]	0.58 [0.004]	0.12 [0.003]	0.11 [0.003]	0.13 [0.003]	0.06 [0.002]
Lower Secondary	0.54 [0.007]	0.12 [0.005]	0.12 [0.004]	0.15 [0.005]	0.07 [0.003]
Higher secondary	0.51 [0.005]	0.14 [0.004]	0.13 [0.004]	0.15 [0.003]	0.07 [0.003]
Tertiary	0.50 [0.006]	0.15 [0.004]	0.15 [0.004]	0.13 [0.003]	0.06 [0.002]
Unknown	0.57 [0.006]	0.14 [0.004]	0.12 [0.004]	0.12 [0.004]	0.04 [0.003]
**Ethnic background**					
Belgian-native[#]	0.49 [0.004]	0.12 [0.002]	0.13 [0.002]	0.17 [0.003]	0.08 [0.002]
West-European	0.50 [0.009]	0.13 [0.006]	0.17 [0.007]	0.14 [0.006]	0.06 [0.004]
South-European	0.54 [0.007]	0.13 [0.005]	0.12 [0.005]	0.15 [0.005]	0.05 [0.003]
East-European	0.59 [0.014]	0.11 [0.009]	0.14 [0.010]	0.12 [0.009]	0.04 [0.006]
Turkish	0.72 [0.008]	0.11 [0.006]	0.07 [0.005]	0.07 [0.005]	0.03 [0.003]
Moroccan/Maghrebian	0.63 [0.005]	0.17 [0.004]	0.11 [0.003]	0.06 [0.002]	0.03 [0.002]
(Non-)Western	0.50 [0.011]	0.16 [0.009]	0.17 [0.009]	0.11 [0.007]	0.05 [0.005]
AIC	132603.9				
BIC	133136.0				
-2 Log Likelihood	132483.906				
Chi-Square	4214.41	(df=56)***			
Pseudo R^2	0.0308				

Note: [a] Model includes educational attainment, parental education and ethnic background; [#] Reference category

Appendix 3 Adjusted predicted probabilities of achieved education, parental education and ethnic background with 95% CIs for the residential mobility types from the baseline multinomial model[a], 2001-2006

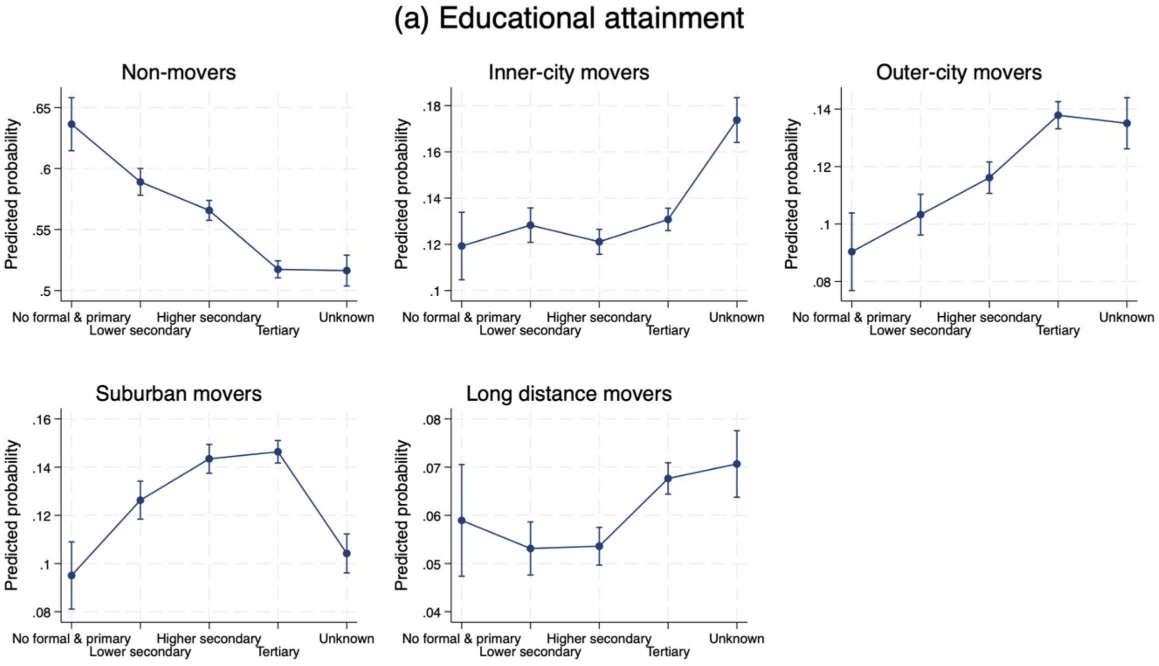

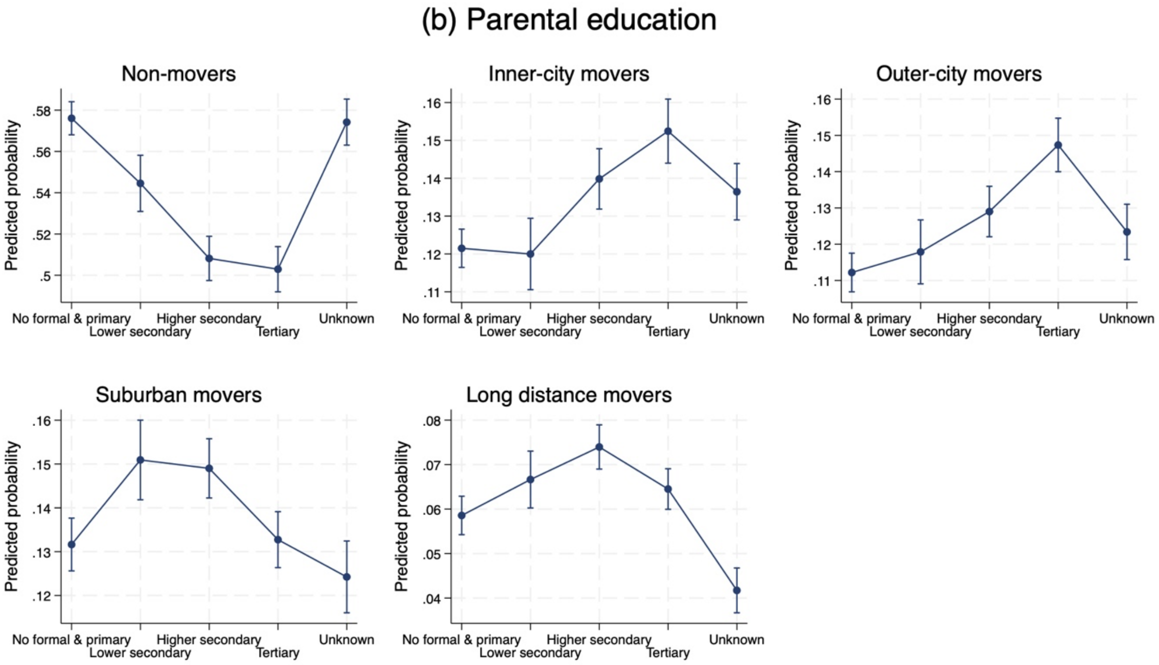

(c) Ethnic background

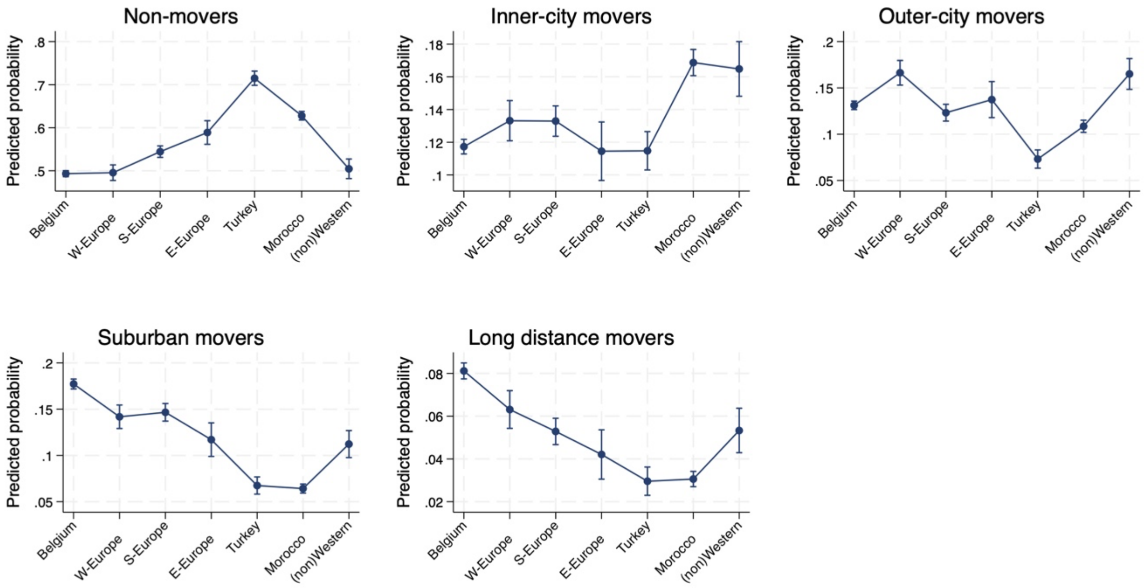

Note: [a] Model includes educational attainment, parental education and ethnic background; The y-scale is not common across residential mobility types due to the varying range in the predicted probabilities.

Appendix 4 Average Marginal Effect (AME) with 95% CIs of educational attainment, parental education and ethnic background for residential mobility types from the extended multinomial model[a], 2001-2006

	Non-Movers	Inner-city movers	Outer-city movers	Suburban movers	Longer-distance movers
	AME [CI]	AME [CI]	AME [CI]	AME [CI]	AME [CI]
**Achieved education**					
No formal – Primary#					
Lower Secondary	-0.04 [-0.08;-0.01]	0.02 [-0.00;0.04]	0.01 [-0.02;0.03]	0.03 [0.01;0.05]	-0.01 [-0.03;0.01]
Higher secondary	-0.04 [-0.07;-0.01]	0.01 [-0.01;0.03]	0.01 [-0.12;0.03]	0.04 [0.02;0.07]	-0.02 [-0.04;0.00]
Tertiary	-0.08 [-0.11;-0.05]	0.02 [0.00;0.04]	0.03 [0.00;0.05]	0.04 [0.02;0.06]	-0.01 [-0.03;0.01]
Unknown	-0.05 [-0.10;-0.01]	0.02 [-0.00;0.05]	0.02 [-0.01;0.05]	0.03 [-0.00;0.06]	-0.02 [-0.04;0.01]
**Parental education**					
No formal - Primary#					
Lower Secondary	0.00 [-0.12;0.02]	-0.01 [-0.02;0.01]	-0.00 [-0.01;0.01]	0.01 [-0.01;0.02]	-0.00 [-0.01;0.01]
Higher secondary	-0.04 [-0.06;-0.02]	0.01 [-0.02;0.02]	0.02 [0.001;0.03]	0.01 [0.00;0.03]	-0.00 [-0.01;0.01]
Tertiary	-0.05 [-0.09;-0.01]	0.02 [-0.01;0.04]	0.04 [0.01;0.07]	-0.01 [-0.02;0.01]	-0.00 [-0.03;0.03]
Unknown	-0.00 [-0.02;0.01]	0.01 [-0.00;0.02]	0.01 [-0.00;0.02]	-0.00 [-0.01;0.01]	-0.02 [-0.03;-0.01]
**Ethnic background**					
Belgian-native#					
West-European	0.00 [-0.02;0.02]	0.11 [0.00;0.03]	0.02 [0.01;0.04]	-0.03 [-0.04;-0.01]	-0.01 [-0.02;-0.00]
South-European	0.00 [-0.02;0.02]	0.02 [0.01;0.03]	-0.00 [-0.02;0.01]	-0.01 [-0.03;-0.00]	-0.00 [-0.01;0.01]
East-European	0.03 [-0.00;0.06]	0.00 [-0.02;0.02]	0.02 [-0.00;0.01]	-0.03 [-0.05;-0.01]	-0.02 [-0.03;-0.01]
Turkish	0.03 [-0.05;0.10]	0.03 [-0.04;0.10]	-0.02 [-0.08;0.04]	-0.09 [-0.12;-0.07]	0.06 [-0.03;0.14]
Moroccan/Maghrebian	0.04 [0.02;0.07]	0.06 [0.04;0.08]	-0.00 [-0.02;0.02]	-0.07 [-0.08;-0.05]	-0.03 [-0.04;-0.02]
(Non-)Western	0.01 [-0.02;0.03]	0.03 [0.02;0.05]	0.02[0.01;0.04]	-0.05 [-0.06;-0.03]	-0.02 [-0.03;-0.00]

Note: [a] Model includes educational attainment, parental education, ethnic background and interactions educational attainment*parental education, educational attainment*ethnic background and parental education*ethnic background, and socio-demographic, socioeconomic and neighbourhood characteristics; # Reference category in multinomial logit model; AME (dy/dx) for factor levels is the discrete change from the base level, indicated with #.

Appendix 5 Average Marginal Effect (AME) with 95% CIs of educational attainment over parental education by ethnic background for each residential mobility type from the extended multinomial model[a], 2001-2006

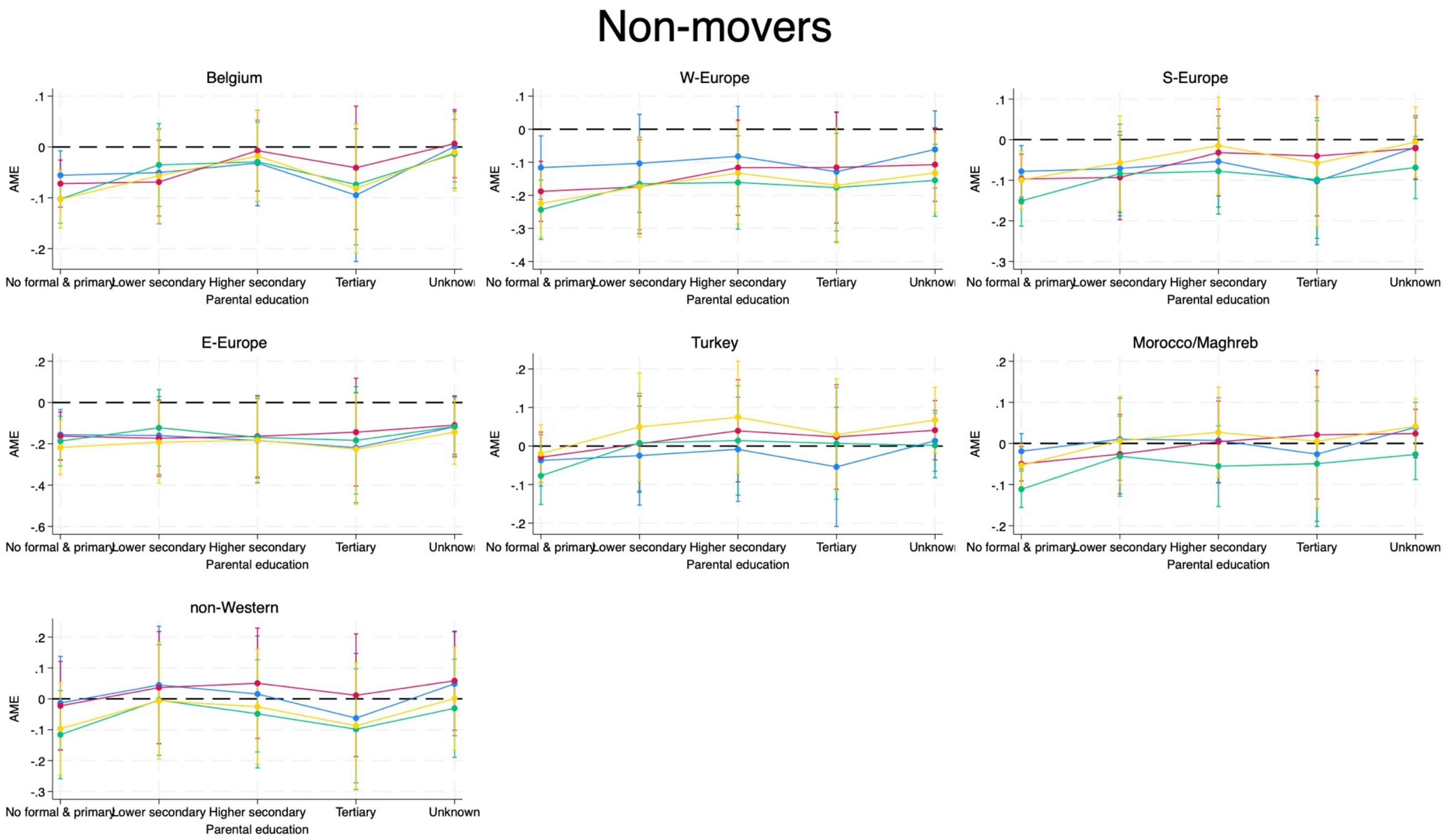

Inner-city movers

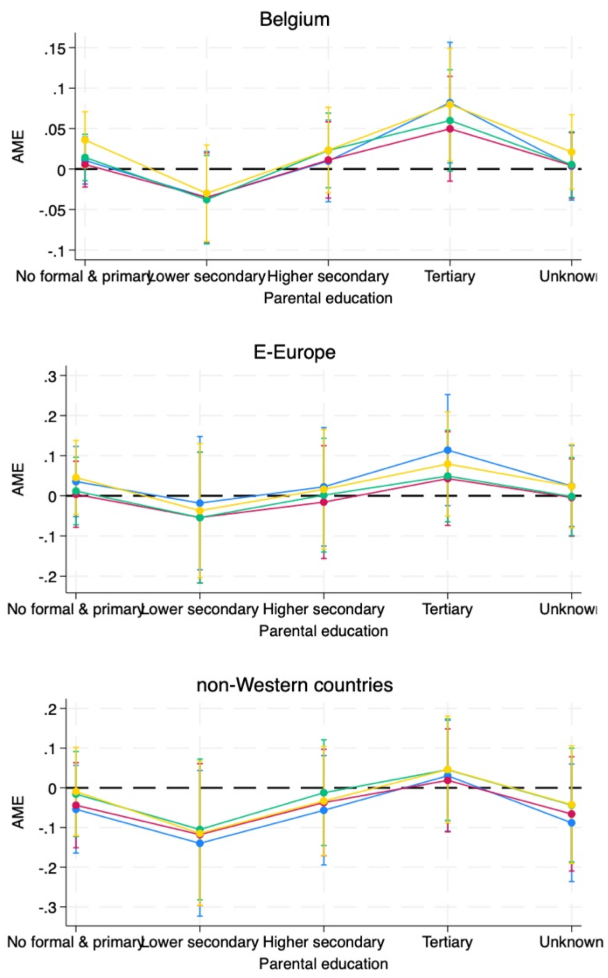

Outer-city movers

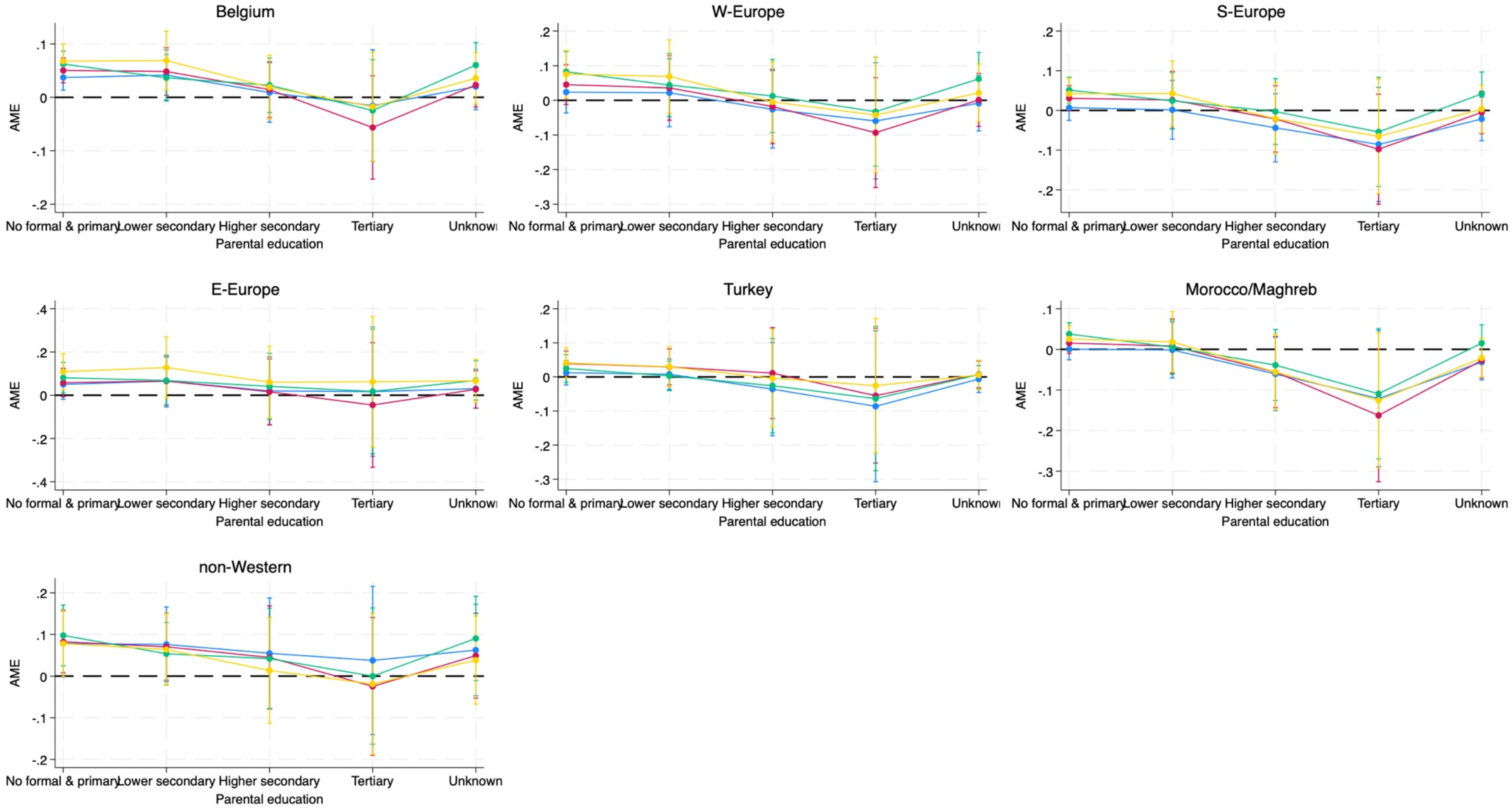

Suburban movers

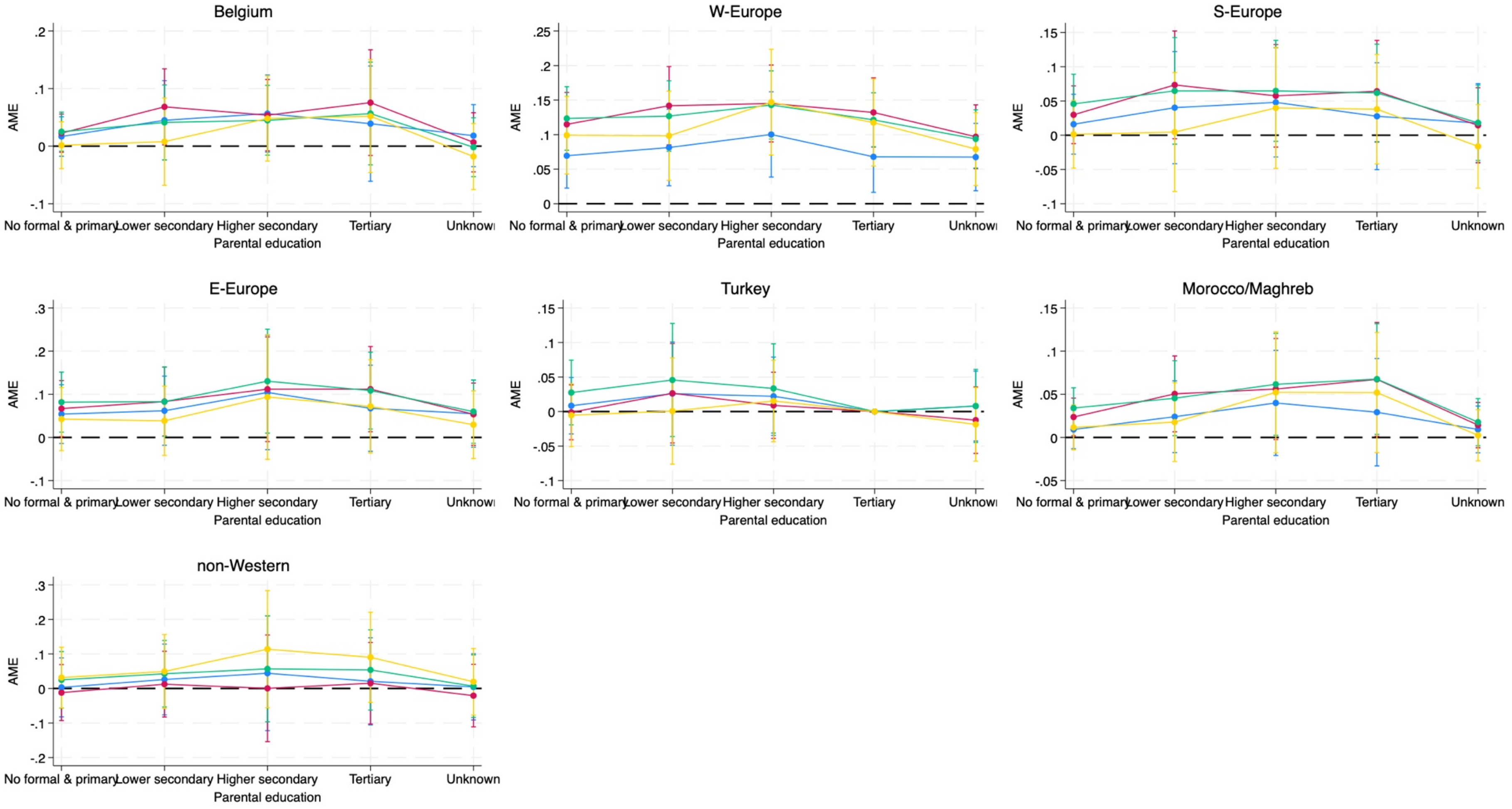

Long distance movers

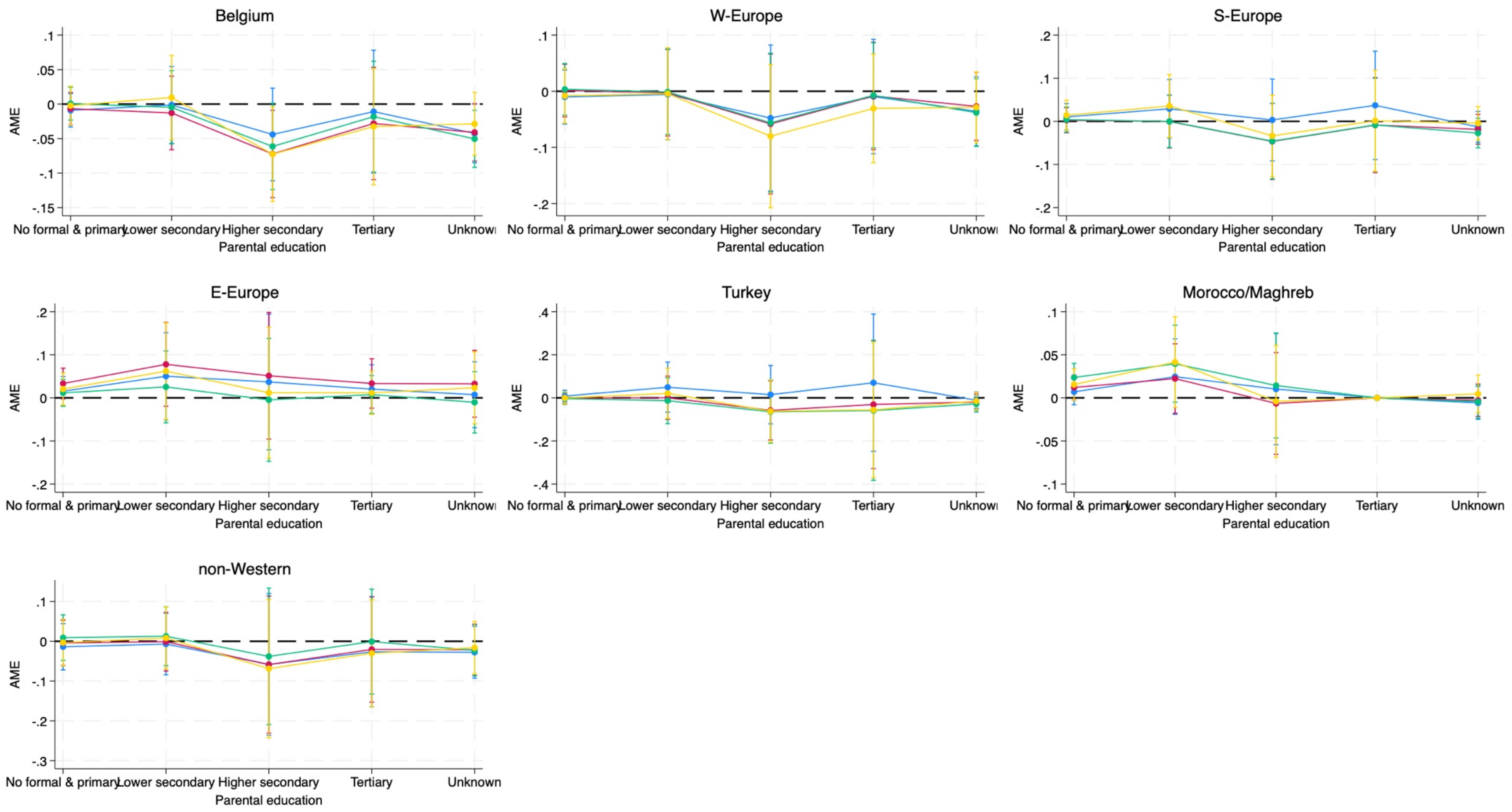

Supplement: Supplementary file 1 — Supplementary file1 (PDF 4599 KB) [file 10680_2023_9690_MOESM1_ESM.pdf]
